# Supplementary figures and images for: Measurement and outcomes of co-production in health and social care: a systematic review of empirical studies
Source: BMJ Open. 2023 Sep 22;13(9):e073808. doi: 10.1136/bmjopen-2023-073808 (PMC10533672; doi:10.1136/bmjopen-2023-073808)

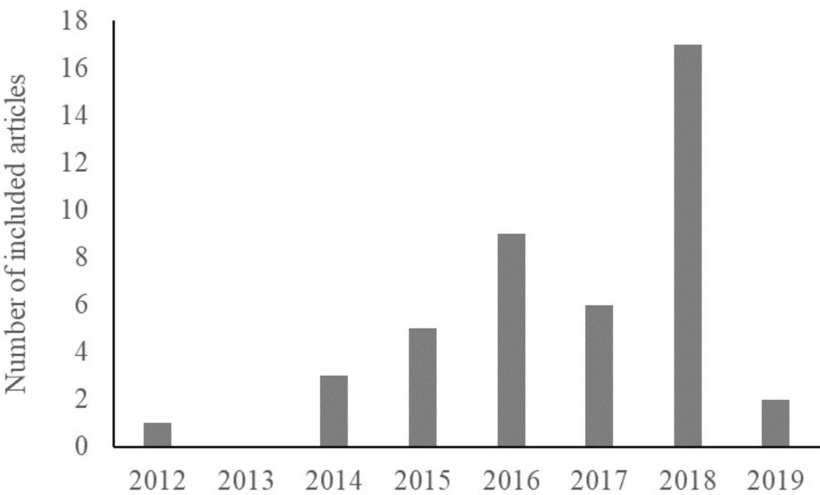

Supplement: Supplementary data [file bmjopen-2023-073808supp003.pdf]
