# Supplementary material for: Measurement and outcomes of co-production in health and social care: a systematic review of empirical studies
Source: BMJ Open. 2023 Sep 22;13(9):e073808. doi: 10.1136/bmjopen-2023-073808 (PMC10533672; doi:10.1136/bmjopen-2023-073808)
Supplement: Supplementary data [file bmjopen-2023-073808supp002.pdf]

Appendix 1. Studies, outcomes and measures.

**Qualitative studies**				
**Paper**	**International validated scales**	**Measures based on publications**	**Self-developed study-specific measures**	**Measures on the experience of co-production**
Tips and Traps: Lessons From Co-designing a Clinician E-Monitoring Tool for Computerized Cognitive Behavioral Therapy [69]				
Shaping innovations in long-term care for stroke survivors with multimorbidity through stakeholder engagement [32]				
Adaption, implementation and evaluation of collaborative service improvements in the testing and result communication process in primary care from patient and staff perspectives: a qualitative study [71]				
Applying social innovation theory to examine how community co-designed health services develop: using a case study approach and mixed methods [70]				
Co-designing for quality: Creating a user-driven tool to improve quality in youth mental health services [72]				

Note Empty fields in the table indicate that no measures were identified. In the qualitative studies, measures are not applicable.

**Quantitative randomized control trials**				
**Papers**	**International validated scales**	**Measures based on publications**	**Self-developed study-specific measures**	**Measures on the experience of co-production**
A Smartphone App (BlueIce) for Young People Who Self-Harm: Open Phase 1 Pre-Post Trial [10]	Strengths and Difficulties Questionnaire (SDQ)<br><br>Mood and Feelings Questionnaire (MFQ)<br><br>Revised Child Anxiety and Depression Scale (RCADS)		The intervention and self-harm	
Digital interventions in severe mental health problems: Lessons from the Actissist development and trial [33]	Positive and Negative Syndrome Scale (PANSS)<br><br>Calgary Depression Scale for Schizophrenia (CDSS)		The intervention and safety	
Who's challenging who training for staff empathy towards adults with challenging behaviour: cluster randomised controlled trial [35]	Maslach Burnout Inventory (MBI)<br><br>Community Living Attitudes Scale (CLAS-MR)<br><br>Staff Positive Contributions Questionnaire (SPCQ)	Staff Empathy for people with Challenging Behavior Questionnaire (SECBO)		
The impact of advertising patient and public involvement on trial recruitment: embedded cluster randomized recruitment trial [34]			Effects of the intervention	

**Quantitative non-randomized controlled studies**				
**Papers**	**International validated scales**	**Measures based on publications**	**Self-developed study-specific measures**	**Measures on the experience of co-production**
Implementing Patient-Oriented Discharge Summaries (PODS): A Multisite Pilot Across Early Adopter Hospitals [36]			Implementation process and intervention functionality	
A quality improvement initiative in community mental health in the republic of Ireland [38]			Experiences of the improvements	
Community organizing and community health: piloting an innovative approach to community engagement applied to an early intervention project in south London [37]	The General Health Questionnaire-12 (GHQ-12)<br><br>The Warwick-Edinburgh Mental Well-being Scale (WEMWBS)	Social Support Program Acceptability Rating Scale (The Treatment Acceptability Rating Scale)<br><br>Functionality of the project (NICE)<br><br>The Adapted Social Capital Questionnaire (World Bank's Social Capital Integrated Questionnaire)	Activities in the program	

**Quantitative descriptive studies**				
**Papers**	**International validated scales**	**Measures based on publications**	**Self-developed study-specific measures**	**Measures on the experience of co-production**
A co-produced self-management programme improves psychosocial outcomes for people living with depression [41]	Patient Activation Measure (PAM)<br><br>Patient Health Questionnaire (PHQ-9)<br><br>Hospital Anxiety Depression Scale (HADS)<br><br>EuroQol<br><br>Health Education Impact Questionnaire (heiQ)			
Impact of a patient-specific co-designed COPD care scorecard on COPD care quality: a quasi-experimental study [42]		Six quality indicators (NICE) matched against data from clinical records	Clinical outcomes of the change	
Health Outcomes and Patient Empowerment: The case of Health budgets in Italy [18]	The Health of the Nation Outcome Scale, Italian version (HoNOS)	Levels of co-production intensity	Costs of a program	
The changing nature of chronic care and co-production of care between primary care professionals and patients with COPD and their informal caregivers [43]	Patient Assessment of Chronic Illness Care questionnaire (PACIC)<br><br>Assessment of Chronic Illness Care, Short version (ACIC-S)			RCS to assess perceptions of interaction productivity
Can an interactive e-learning training package improve the understanding of personality disorder within mental health professionals? [40]		The Personality Disorder-Knowledge, Attitudes and Skills Questionnaire (PD-KASQ)		

		Efficiency of intervention (researchers' earlier study)		
Okay to Stay? A new plan to help people with long-term conditions remain in their own homes [39]			Efficiency of the program	

**Mixed method studies**				
**Papers**	**International validated scales**	**Measures based on publications**	**Self-developed study-specific measures**	**Measures on the experience of co-production**
Improving healthcare through the use of co-design [47]		Experiences of program and number of ideas. (NHS survey)		
An evaluation of Knowledge and Understanding Framework Personality Disorder Awareness Training: Can a co-production model be effective in a local NHS mental health Trust? [52]		The Personality Disorder-Knowledge, Attitudes and Skills Questionnaire (PD-KASQ)	Effects of the program	
Consumer measures and research co-production: A pilot study evaluating the recovery orientation of a mental health program collaboration [48]	The Recovery Self-Assessment (RSA)<br><br>The Recovery Enhancing Environment measure (REE)			
The use of a co-design model in improving timely bleed reporting by adults with haemophilia living in the Auckland region of New Zealand [61]		Mean bleed reporting time<br><br>Bleeds per month<br><br>Bleeding episodes outside of a prespecified 48-h	The clinical effects of the program	
Evaluating 'FREDA Challenge': A co-produced human rights board game in services for people with intellectual disabilities [53]		Human right quiz<br><br>Attitudes to Human Rights questionnaire (ATHIRI)	The attitudes, knowledge, and opinions of the intervention	
Evaluating a novel cervical orthosis, the Sheffield Support Snood, in patients with amyotrophic lateral sclerosis/motor neuron disease with neck weakness [60]			The experiences, comfort and satisfaction with the intervention	
Evaluating the Prosper peer-led peer support network: A participatory, coproduced evaluation [63]			The experiences and priorities on the service	

Mobile internet service for self-management of physical activity in people with rheumatoid arthritis: evaluation of a test version [59]			The innovation, self-monitoring and feedback	
Promoting physical activity among children and youth in disadvantaged South Australian CALD communities through alternative community sport opportunities [44]			The thoughts and feelings of the project	
Enhancing the experience of carers in the chemotherapy outpatient setting: an exploratory randomised controlled trial to test impact, acceptability and feasibility of a complex intervention co-designed by carers and staff [58]	The General Health Questionnaire-12 (GHQ-12)<br><br>Supportive Care Needs Survey for Partners and Carers [SCNS-P&C)	Perceived confidence in supporting friend or relative	The effects of the intervention; knowledge and experiences of care	
Improving Patient-Centered Care for Young People in General Practice With a Co-designed Screening App: Mixed Methods Study [55]	Mobile Application Rating Scale (MARS)	Patient-centered care<br><br>Disclosure<br>Fear of judgment<br>App acceptability<br><br>Knowledge, confidence and enthusiasm<br><br>Youth friendly care	The opinions and willingness to use the innovation	
Integrating Engagement and Improvement Work In a Paediatric Hospital [50]			Impressions and feelings about integrating EBCD and quality improvement tools	
Which green way: description of the intervention for mobilising against Aedes aegypti under difficult security conditions in southern Mexico [45]		Clinical indicators<br><br>Proved infections	Self-reported health outcomes	
Impact evaluation of an Independent Mental Health Advocacy (IMHA) service in a high secure hospital: a co-produced survey measuring self-reported changes to patient self-determination [62]			The improved services' relevance, accessibility and ease of use	

Improving the quality and content of midwives' discussions with low-risk women about their options for place of birth: Co-production and evaluation of an intervention package [64]			The level of knowledge and confidence regarding safety and intervention rates, views of the intervention package<br><br>Objective knowledge calculated using multiple-choice questions	
Design and development of a gait training system for Parkinson's disease [51]	System Usability Scale (SUS)		The development and improvement of a customer focused innovation	
Patient safety after implementation of a co-produced family centered communication programme: multicenter before and after intervention study [54]		Medical errors<br><br>Harmful errors<br><br>Adverse events	Family experiences	
Co-design of an evidence-based health education diabetes foot app to prevent serious foot complications: a feasibility study [67]	The Nottingham Assessment of Functional Footcare (NAFF)	Diabetes foot care knowledge<br><br>Attitudes		
Evaluation of a peer support specialist led group [66]			The experiences and effects of the recovery group<br><br>The number of referrals and contacts	
Patient-reported safety incidents as a new source of patient safety data: an exploratory comparative study in an acute hospital in England [68]			The number of safety incidents counted and compared by methods	
Using Implementation Intentions to Prevent Relapse after Psychological Treatment for Depression - the SMArT Intervention [65]	Patient Health Questionnaire (PHQ-9)<br><br>Generalized Anxiety Disorder Questionnaire (GAD-7)		Counts of self-regulatory strategies, IMPS	

Development of a patient-centred intervention to improve knowledge and understanding of antibiotic therapy in secondary care [57]		Patient knowledge and understanding surrounding antimicrobial therapy and resistance		
Co-Designing an eHealth Service for the Co-Care of Parkinson Disease: Explorative Study of Values and Challenges<br>[46]			The experiences and views of the workshops and logistics	Experience: Questionnaire on participants' overall experiences of participation
Knowledge Is Power: Improving Outcomes for Patients, Partners, and Professionals in the Digital Age [49]	Glasgow Hearing Aid Benefit Profile (GHABP)<br><br>The International Outcome Inventory for Hearing Aids (IOI-HA)<br><br>The Hearing Handicap Inventory for the Elderly (HHIE)		Patient feedback on how they experienced the innovation	
Community-based participatory action research on rheumatic heart disease in an Australian Aboriginal homeland: Evaluation of the 'On track watch' project [56]		Values and Ethics Guidelines, National Health & Medical Research Council<br><br>'How-to' guide, National Health Medical Research Council		Emoticon survey based on National Health and Medical Research Council principles<br>Reciprocity, respect<br>Equality<br>Responsibility<br>Survival and Protection<br>Spirit & integrity
