# Supplementary material for: Measurement and outcomes of co-production in health and social care: a systematic review of empirical studies
Source: BMJ Open. 2023 Sep 22;13(9):e073808. doi: 10.1136/bmjopen-2023-073808 (PMC10533672; doi:10.1136/bmjopen-2023-073808)
Supplement: Author's manuscript [file bmjopen-2023-073808.draft_revisions.pdf]

BMJ Open

BMJ Open is committed to open peer review. As part of this commitment we make the peer review history of every article we publish publicly available.

When an article is published we post the peer reviewers' comments and the authors' responses online. We also post the versions of the paper that were used during peer review. These are the versions that the peer review comments apply to.

The versions of the paper that follow are the versions that were submitted during the peer review process. They are not the versions of record or the final published versions. They should not be cited or distributed as the published version of this manuscript.

BMJ Open is an open access journal and the full, final, typeset and author-corrected version of record of the manuscript is available on our site with no access controls, subscription charges or pay-per-view fees (http://bmjopen.bmj.com).

If you have any questions on BMJ Open's open peer review process please email info.bmjopen@bmj.com

BMJ Open

The measurement and outcomes of co-production in health and social care - a systematic review of empirical studies

Journal:	*BMJ Open*
Manuscript ID	bmjopen-2023-073808
Article Type:	Original research
Date Submitted by the Author:	20-Mar-2023
Complete List of Authors:	Nordin, Annika; Jönköping University School of Health and Welfare, Jönköping Academy of Improvement of Health and Welfare<br>Kjellstrom, Sofia; Jönköping University School of Health and Welfare, Jönkoping Academy of Improvement of Health and Welfare<br>Robert, Glenn; King's College London, Florence Nightingale Faculty of Nursing, Midwifery & Palliative Care,<br>Masterson, Daniel; Jönköping University School of Health and Welfare, Jönköping Academy for Improvement of Health and Welfare<br>Areskoug Josefsson, Kristina; Högskolan i Trollhättan, Department of Health Sciences; Jönköping University School of Health and Welfare, Jönköping Academy of Improvement of Health and Welfare
Keywords:	Patient Participation, Quality in health care < HEALTH SERVICES ADMINISTRATION & MANAGEMENT, Systematic Review

SCHOLARONE™ Manuscripts

BMJ

I, the Submitting Author has the right to grant and does grant on behalf of all authors of the Work (as defined in the below author licence), an exclusive licence and/or a non-exclusive licence for contributions from authors who are: i) UK Crown employees; ii) where BMJ has agreed a CC-BY licence shall apply, and/or iii) in accordance with the terms applicable for US Federal Government officers or employees acting as part of their official duties; on a worldwide, perpetual, irrevocable, royalty-free basis to BMJ Publishing Group Ltd ("BMJ") its licensees and where the relevant Journal is co-owned by BMJ to the co-owners of the Journal, to publish the Work in this journal and any other BMJ products and to exploit all rights, as set out in our licence.

The Submitting Author accepts and understands that any supply made under these terms is made by BMJ to the Submitting Author unless you are acting as an employee on behalf of your employer or a postgraduate student of an affiliated institution which is paying any applicable article publishing charge ("APC") for Open Access articles. Where the Submitting Author wishes to make the Work available on an Open Access basis (and intends to pay the relevant APC), the terms of reuse of such Open Access shall be governed by a Creative Commons licence – details of these licences and which Creative Commons licence will apply to this Work are set out in our licence referred to above.

Other than as permitted in any relevant BMJ Author's Self Archiving Policies, I confirm this Work has not been accepted for publication elsewhere, is not being considered for publication elsewhere and does not duplicate material already published. I confirm all authors consent to publication of this Work and authorise the granting of this licence.

For peer review only

The measurement and outcomes of co-production in health and social care - a systematic review of empirical studies

Abstract

Background*:* Co-production is increasingly promoted as an effective way of improving the quality of health and social care services but the diversity of measures used in individual studies makes their outcomes difficult to interpret.
Objective: The objective is to explore how empirical studies in the health and social care context have described the outcomes of co-production projects and how those outcomes have been measured.
Design and methods: A wider scoping review forms the basis for this systematic review. Search terms for the concepts (co-produc* OR coproduc* OR co-design* OR codesign*) and contexts (health OR 'public service* OR "public sector") were used in the following databases: CINAHL with Full Text (EBSCOHost), Cochrane Central Register of Controlled trials (Wiley), MEDLINE (EBSCOHost), PsycINFO (ProQuest), PubMed (legacy) and Scopus (Elsevier). This systematic review is reported according to the PRISMA guideline. The Mixed Methods Appraisal Tool underpinned the quality of included papers.
Results: 43 empirical studies were included. They were conducted in 12 different countries, with the UK representing more than 50 % of all papers. No paper was excluded due to the Mixed Method Quality Appraisal screening and 60 % of included papers were mixed method studies. The extensive use of self-developed study-specific measures hampered comparisons and cumulative knowledge-building. Overall, the studies reported positive outcomes. Co-production was reported to be positively experienced and provided important learning.
Conclusions: The lack of common approaches to measuring co-production is more problematic than the plurality of measurements itself. This systematic review suggest that co-production should be measured from three perspectives: outputs of co-production processes, the experiences of participating in co-production processes, and outcomes of co-production. Both self-developed study-specific measures and established measures should be used. The maturity of this research field would benefit from the development and use of scientific reporting guidelines.

Article summary

Strengths and limitations of the study

This systematic review embraces co-production within both health and social care, which facilitates a holistic understanding of co-production from a user perspective.

This systematic review regards empirical studies, indicating that identified measures are relevant and possible to use practically, in empirical settings.

Only peer reviewed research published in English is included, which may mean that important co-productions research published in other languages has been overlooked.

Key words: co-production, patient participation, quality in healthcare, systematic review

For peer review only

The measurement and outcomes of co-production in health and social care - a systematic review of empirical studies

Introduction

Despite the term co-production stemming back to Ostrom's seminal work in the 1970s [1], the study of co-production is nonetheless sometimes viewed today as having low scientific maturity [2-4]. For the purposes of this review – and following others [5]- we include co-design with its origins in the participatory design movement in Scandinavia also in the 1970s as a specific form of co-production. Co-production research has been reported as typically not outcome-focused, tending rather to describe co-production processes [3, 6]. The concepts of co-production and co-design are attracting increasing research interest [2, 7], albeit whilst appearing to labor under a multitude of definitions [7]. This systematic review adopts the definition proposed by Osborne et al.; 'the voluntary or involuntary involvement of public service users in any of the design, management, delivery and/or evaluation of public services' [5, p 640].

This systematic review is part of the Samskapa research program on co-production [8] and concerns the measurement and outcomes of co-production as it relates to the provision of both health and social care services. Health and social care are provided by various organisational types in different countries, including private, public and third sector organisations. Such services aim to improve social or health-related aspects of citizens' lives and are important for quality of life [9]. The increasing number of empirical studies on co-production in these sectors are predominantly in health care; of the ten most cited papers on co-production, none concern social care [2]. Given widely held ambitions to integrate health and social care services [9], this focus is problematic, and thus, this systematic review embraces both health and social care services.

Advocated as an effective way of improving quality of health and social care [10-12], co-production is also considered more broadly as a means to promote a democratic ethos placing equal value on the contributions of both citizens and service providers in the successfully delivery of services [11, 13]. The benefits of Patient and Public Involvement (PPI) in research is also acknowledged as a moral obligation with several potential benefits [14].

There are several plausible explanations related to the challenges researchers encounter when seeking to measure the outcomes of co-production [15], including its emergent and adaptive nature which can impede the early determination of appropriate measurements and which might otherwise neglect outcomes that are significant to participants [16]. Co-production also often encompasses complex and broad needs [17], and is characterized by both a multitude of stakeholders and a plurality of contexts; such features tend to lead to weak or indistinct cause-effect relationships between co-production activities and their outcomes [18]. However, knowledge of co-production outcomes is important in order to enable the continued improvement of services [19]. In summary, whilst important to explore the outcomes of co-production, these are often difficult to measure through a priori measures and deductive analysis [6, 20, 21], and to date methodological diversity has been limited [12].

For peer review only

Regardless of these challenges, scholars have previously used various categorisations to present outcomes, further complicating attempts to synthesize them and build a broader evidence base. Examples of categories of co-production outcomes include: outputs and outcomes [10], discrete products, care processes and structural outcomes [22], patient/staff involvement, generating ideas and tangible change [23] and both individual and collective outcomes [4]. Alternatively, innovation potential, individual well-being and citizen empowerment, increased effectiveness and efficiency, mobilization of resources and increased democracy are categories suggested by Brix et al. [18]. Other types of similar broader outcomes include increasing effectiveness, increasing citizen involvement, greater efficiency, gaining customer satisfaction, strengthening social cohesion and democratizing public services [3].

There are a small number of reviews appraising the research designs and methods of co-production across the public sector. In their systematic review on empirical studies on co-production and co-design e.g., Voorberg and colleagues report that qualitative case studies, single or comparative, predominate [3]. More robust critiques of the designs and methods typically employed to study the outcomes of co-production may help to improve the scientific maturity of this research field and to enable better understanding of the wide range of reported outcomes. In this systematic review, outcomes are understood as qualitative and quantitative descriptions used to capture the impact, effect, results, or outputs of ongoing, or implemented, co-production projects. Objectives, measures and outcomes are interrelated. Outcomes are described by the use of measures, and evaluated in relationship to stated objectives. In seeking to explore the measures and outcomes of co-production, knowledge of the project objectives is necessary.

The aim of this systematic review is to explore how empirical studies in health and social care have described the outcomes of co-production projects and how such outcomes have been measured.
The review questions are:

- What are the objectives of co-production projects?
- What measures have been used?
- What are the outcomes of co-production?

Methods and materials

In the published Samskapa study protocol the aims were to explore, enhance and measure the value of co-production for improving the health and social care of citizens [8]. A scoping review was performed as part of this wider study in March 2019 [7], and the results form the basis for this systematic review. This systematic review has followed the Preferred Reporting Items for Systematic Reviews and Meta-Analyses, the PRISMA guidelines [24], and the manuscript is organised according to the instructions for authors.

Information sources, eligibility criteria, search strategy and selection process for the scoping review

The scoping review set out to explore 'what is out there' in the peer reviewed research relevant to co-production or co-design in health and social care services. (See online supplemental file 1, for full search strategy). In brief, search terms for the concepts (co-

For peer review only

produc* OR coproduc* OR co-design* OR codesign*) and contexts (health OR 'public service* OR "public sector") were used in the following databases: CINAHL with Full Text (EBSCOHost), Cochrane Central Register of Controlled trials (Wiley), MEDLINE (EBSCOHost), PsycINFO (ProQuest), PubMed (legacy) and Scopus (Elsevier). There was no specified date limit. Articles were included in the scoping review if they related to peer-reviewed research, consisted of any methodology relevant to co-production or co-design in the context of health and social care services, involving service-users and were written in English. The results were stored in a database in Excel and in Rayyan, a web- and mobile app for systematic reviews that helps expedite the initial screening of abstracts and titles using a semi-automation [25].

Eligibility criteria and selection process for this systematic review

The results from the scoping review were screened initially by one of the researchers (D.M), to remove papers without empirical data or explicit references to measurements. The titles and abstracts of identified papers were added into a new Rayyan data base and reviewed by two of the researchers (AN and KAJ), who independently noted 'include', "unsure" or 'exclude' in Rayyan based on the three eligibility criteria for this systematic review:

1. Process of co-production is described (inclusion criterion)
2. Original data from process and outcomes is described (inclusion criterion).
3. Not a theoretical, conceptual, protocol, review or psychometric paper (exclusion criterion)

Conflicting decisions, or decisions the researchers were undecisive of, were crosschecked and validated by the two researchers and remaining conflicts were discussed until consensus was reached. The eligibility criteria were then applied on the included full text papers. To enhance the reliability of the review process, the two researchers read the same first five papers and discussed their decisions until consensus was reached. Another five papers were reviewed following the same procedure and thereafter, remaining papers were examined separately, with decisions noted in a shared Excel file. The quality of each included papers was then appraised.

The Mixed Method Quality Appraisal Tool (MMAT) has been developed to assess the methodological quality of empirical studies [26] and was used to appraise the quality of included full text papers. MMAT includes five study designs (qualitative, randomized controlled trial, non-randomized, quantitative descriptive and mixed methods) and for each study design, five criteria for the evaluation of the methodological quality are provided. [27]. The criteria can be evaluated 'yes', 'no' or 'can't tell', and affirmative responses indicate quality. Two initial screening questions are included to assess whether the study is empirical. The first five papers were assessed jointly by two researchers (AN and KAJ). Another five papers were assessed separately and the results compared and discussed. On the basis of the MMAT tool, the remaining papers were assessed independently and the results documented in a shared Excel file. Two meetings of the researchers were held to discuss queries. The MMAT study designs are used as categories to group the included studies in the synthesis of our findings below.

For peer review only

Data collection process and data items for this systematic review

To develop a shared work procedure for the data collection two researchers (AN and KAJ) jointly extracted data from five papers and compared and discussed the results. To assess the risk of bias the researchers discussed the chosen data collection methodology and examples of the first findings with PhD students in the Samskapa research program. The remaining data extraction was carried out independently by the two researchers and documented in separate files, one for each MMAT category. To enhance consistency, data extraction definitions were inserted as column headings in the Excel files. A count of the number of papers within each MMAT study design category was made. A count was also completed for each of the study design characteristic variables and the proportion of affirmative responses on the MMAT screening questions. After completion of the data extraction, the findings were again discussed with PhD students in the Samskapa research program. A plain language blogpost including discussion points inviting comments from a wider audience was also written [28].

In Table 1, the research questions are presented alongside the definitions for data extraction.

Table 1. Research questions and data extraction definitions.

**Research questions**	**Data extraction definitions**
What are the objectives for co-production projects?	Descriptions of the initiators of the projects and the aims of the studies.
What measures are used?	Descriptions of used measures (scales, questionnaires or other forms of measurements).
What outcomes of co-production are reported?	Qualitative or quantitative descriptions of the impact, effects, results or outputs of ongoing or implemented co-production projects, including learning and social and cultural effects.

In Table 2, other variables for which data was sought are listed and defined.

Table 2. Variables for study characteristics and data extraction definitions.

**Study characteristics**	**Data extraction definitions**
Year	Year of publication
Country	Country in which the study was conducted
Field	The specific health- or social care field for the study

Synthesis methods

The extracted data in each MMAT study design category was analysed separately. In the first step of analysis, the data extractions were closely read. In the next step, the data extractions formed the basis for a convergent qualitative synthesis [27]. A synthesis is a process of putting the findings from individual studies together into a new or different arrangement and thus providing knowledge that would not otherwise not apparent while reading the individual studies in isolation [29]. The measures were analysed and coded into four categories 1) International validated scales (including scales with minor adaption or use of subscales), 2) Measures based on publications (e.g. based on public

documents, published research, indicators and non-validated scales), 3) Self-developed study-specific measures (locally developed measures), and 4) Measures on the experience of co-production.

Results of the systematic review

Study selection

The first initial screening of all papers included in the scoping review reduced the number of potential papers from 979 to 187. Thereafter the titles and abstracts of the remaining papers were reviewed and 116 papers were excluded. Thirty-seven papers were excluded based on the first eligibility criteria, 34 papers based on the second eligibility criteria and 45 based on the third eligibility criteria. The eligibility criteria were then applied on the included full text papers, leading to the exclusion of a further 28 papers. Fifteen papers were excluded based on the first eligibility criteria, 8 papers based on the second eligibility criteria and 5 based on the third eligibility criteria. The remaining 43 papers formed the basis for the quality appraisal and the data extraction for this systematic review. The PRISMA flow diagram is based on the PRISMA 2020 flow diagram template for systematic reviews [30], and is presented in Figure 1.

Figure 1. The PRISMA flow diagram
Please insert figure 1 here

Result of the quality assessment

Of all included papers in the systematic review, 86% were considered as having a clear research question (screening question No 1 of MMAT) and 95% to have a relevant data collection process (screening questions No 2 of MMAT). The largest proportion of included papers (60%) reported on studies with mixed method study designs (Table 3). The distribution of affirmative responses to the MMAT categories indicate the general high quality of the included studies.

Table 3. Proportion of affirmative categories in MMAT per study design.

	Yes on 5 MMAT criteria	Yes on 4 MMAT criteria	Yes on 3 MMAT criteria	Yes on 2 MMAT criteria	Yes on 1 MMAT criteria	Yes on 0 MMAT criteria
**Qualitative studies** 12 % (5)	60 % (3)	0 % (0)	40 % (2)	0 % (0)	0 % (0)	0 % (0)
**Quantitative randomized controlled trials** 7 % (4)	25 % (1)	0 % (0)	25 % (1)	25 % (1)	25 % (1)	0 % (0)
**Quantitative non-randomised studies** 7 % (3)	0 % (0)	33.3% (1)	33.3 % (1)	0 % (0)	0 % (0)	33.3 % (1)
**Quantitative descriptive studies** 14 % (6)	50 % (3)	33 % (2)	0 % (0)	17 % (1)	0 % (0)	0 % (0)

For peer review only

**Mixed methods studies** 60 % (25)	40 % (10)	24 % (6)	16 % (4)	12% (3)	8 % (2)	0 % (0)

Study characteristics

The studies located in this review were published between 2012 and 2019 (Figure. 2). The year 2018 saw the most papers (n=17), however only the first months of 2019 are included, see online supplemental figure 2.

The 43 studies were conducted in 12 different countries, generally with three or fewer studies per country. The exceptions are the United Kingdom (n=23) and Australia (n=6). The studies were conducted in different fields of health and social care; mental health has the largest proportion of studies (n=14). The UK represents 53% of the included studies and of the 23 studies conducted there, 9 related to a mental health setting.

Based on our convergent design [27] our findings are reported below for each research question in turn. In the text, user is used as a generic term for those who are using a service, innovation or healthcare innovation in health and social care. The term informal caregiver is used as a generic term for individuals supporting and helping the users, based on personal engagement (and not a professional responsibility) and providers is used as a generic term for those who are providing a service. In online supplemental Appendix 1, the outcomes and measures are compiled.

Results of syntheses

What are the objectives of co-production projects?

The objectives were explored by analysis of who initiated the co-production projects and establishing the aims of the study. Users rarely initiated co-production projects or studies.

The aims of the *qualitative studies* ranged from increasing communication with users to encouraging service innovation in partnership with them. Some of the studies also aimed to increase users' engagement with health and social care services and to assess how users and providers perceive services and experience quality [31]. In the few cases the origins of the studies were described, the studies had been initiated by researchers.

All the *quantitative randomized controlled trials* aimed to evaluate outcomes of co-produced interventions. Two studies aimed to provide better, more timely support through the use of digital tools [32, 33]. One study aimed to increase the patient and public involvement in research [34] and a further study aimed at improving staff behaviours and actions to improve care through co-produced interventions [35]. Those who initiated the projects - and their reasons for doing so - were not described in the studies.

Descriptions of the initiators of project were only included in two of the three *quantitative non-randomized controlled studies*. The project by Hahn-Goldberg et al. [36] was initiated by the hospital management after a co-produced pilot study and the project by Bolton et al. [37] was initiated by several organisations aiming to promote

For peer review only

public health. All three studies aimed at improving users´ experiences of provided services [36-38].

None of the included *quantitative descriptive studies* described users as having initiated the projects. Chapman et al. [39] aimed at evaluating a co-produced plan to reduce emergency visits. Lamph et al. [40] aimed at evaluating co-production in an e-learning program to improve staff attitudes. Turner et al. [41] aimed at evaluating a co-designed and co-produced program to improve patients' health and self-management skills and Roberts et al. [42] aimed at improving care through individualized care quality data. Some studies aimed at increasing co-production within processes. Cramm et al. [43] aimed at developing the care process by improving the co-productive relationship. Adinolfi et al. [17] aimed to improve co-production processes and patient outcomes by integrating health and social care and increasing user empowerment.

The aims of the *mixed methods studies* varied. One study aimed to empower disadvantaged communities [44] and one aimed at testing co-production in areas with high levels of violence [45]. Other aims were to evaluate the experience of part-taking in co-design [46] and to evaluate how co-design can improve patient experiences and services [47]. Four studies aimed at improving co-production within already ongoing work processes [48-51]. Most of the studies aimed at evaluating outcomes of co-produced information, interventions/services or products [47, 48, 52-67]. Among the studies evaluating outcomes of co-produced interventions/services the focus was on effectiveness [52, 61] and on the level of improvement [54]. Evaluation of acceptability and feasibility of co-produced interventions or products was the aim of several studies [58, 65, 67] and one study aimed at evaluating the utilization and experience of a co-designed product [59]. Other studies aimed at evaluating user-led co-produced interventions [63, 66]. However, of the 25 mixed method studies only one stated that the study was initiated by users [56]. Also, only one study compared a co-produced method with other methods [68].

What measures are used?

In the *qualitative studies*, no measures beyond simple descriptive calculations were used.

In the *quantitative randomized controlled trials* several validated scales were used. Two studies used three different validated scales each [32, 35] and one [33] used two validated scales. The scales focused on health- or clinical aspects related to the interventions. Two self-developed study-specific measurements purposing to evaluate the intervention itself were used [32, 34, 35] and in the study by Hasting et al. [35] a scale based on previous publications was used.

Self-developed study-specific measurements focusing on the experiences of an intervention and the implementation process were used in the *quantitative non-randomized controlled* studies [36-38]. In the study by Bolton et al. [37] two validated scales focusing on health aspects related to the intervention and three questionnaires based on other publications were also used. It is worth noting that the validity and reliability of the outcome measurements were rarely discussed. The focus was more on the sustainability and usefulness of the innovations/interventions.

In three of the six *quantitative descriptive studies* validated scales were used. Turner et al. [41] used five different validated scales. Cramm et al. [43] one study used two validated scales and Adinolfi et al. [17] used one validated scale. The scales focused on health or clinical aspects related to the interventions. Two studies included self-developed study-specific measurements concerning the interventions [17, 39] and one study included self-developed measurements regarding the clinical outcomes [42]. Three studies used measurements based on previous publications [17, 40, 42]. Cramm et al. [43] used The Relational Coordination Scale (RCS) to assess participants' perceptions of interaction productivity, thus measuring the experience of the co-production process itself.

Seven of the 25 *mixed method studies* used validated scales. In three studies, one validated scale was used [51, 55, 67]. Three studies used two different validated scales [48, 58, 65] and Ferguson et al. [49] used three different validated scales. Two of the studies used scales that, unlike the others, did not focus on health or clinical aspects. Instead, they used validated scales measuring the quality [55] and usability of technical systems or innovations [51]. Eleven studies used one or more measures based on previous research [45, 47, 52-58, 61, 67]. Five studies did not use any self-developed study-specific measures [47, 48, 56, 57, 67]; all others did. Two studies used measures regarding the experience or level of the co-production process. Revenäs et al. [46] used a self-developed survey to assess users' experience of participating in the co-production process, and Haynes et al. [56] measured how the intervention adhered to the National Health and Medical Research Council's (NHMRC) principles. Brosseau et al. [50] did not measure the level of co-production but referred to several ways this could have been done. Morales-Peres et al. [45] also did not measure the co-production level; however, they concluded that the study results were made possible due to the high levels of participatory engagement.

What outcomes of co-production are reported?

A wide variety of outcomes were presented in the *qualitative studies,* commonly describing the number of innovations or implemented changes, the establishment of priority areas or consensual agreement and the development of a tool [31, 69-72]. The sustainability of outcomes was presented as having to encompass financial sustainability, new organisational relationships and further quantitative studies [70]. Organisational structures and issues related to patient safety were described as potentially hampering the implementation of digital tools, thus influencing sustainability negatively [31].

The outcomes in the *quantitative randomized controlled trials* were positive for two interventions targeting users, showing significant reduced self-harm and reduced mental ill-health, and that the users wanted to continue using the co-produced digital tools [32, 33]. A third study targeting mental health patients did not report any positive outcomes [34]. The outcomes for interventions targeting providers were also favourable, but the results were not as strong as in the studies targeting users [35].

Regarding the *quantitative non-randomized controlled studies,* Hahn-Goldberg et al. [36] presented outcomes related to the use of a new tool, but the effects of the tool, including the costs are yet to be evaluated. With the objective of identifying the vital outcomes of the project, Murphy et al. [38] used several measurements covering different

For peer review only

perspectives. The measurements concerned outcomes of the process itself, user surveys and user narratives. Some of the surveys aimed at family members had response rates too low to provide useful data. However, narratives from users were used to complement the survey data and these data showed both positive and sustainable outcomes. The research teams concluded that the value of collaborating with stakeholders to find useful measurement and tools for assessments was an important learning outcome. Bolton et al. [37] used measures not tested beyond their face validity but there were other outcomes showing positive results such as new social support networks and user requests for support in gaining knowledge supporting their health.

Cramm et al. [43] and Lamph et al. [40] described quantitative results and correlations from established questionnaires in their *quantitative descriptive studies.* However, Lamph et al. [40] also included observational process outcomes. Turner et al. [41] additionally included health data, registry data and observational process outcomes. Chapman et al. [39] described quantitative results of objective measurements and Roberts et al. [42] described compliance with clinical practices and quality indicators. Quantitative outcomes in terms of individual health status and cost savings on organisational levels were also reported by Adinolfi et al. [17].

The outcomes in the *mixed method studies* were extensive and to facilitate an overview the outcomes are presented below in four broad themes: effects on the outcomes and processes, success factors, impact on participants and learning outcomes.

Outcomes and processes

Co-production led to many effects on the outcomes and processes the projects aimed to improve, with improved care [62], stronger social networks [63] and increased urgency in communication as [56] some examples. Co-production with users indicated what worked well and what needed to be improved [48, 66] and combining quality improvement and co-design led to additional quality improvement projects [50]. Co-produced products like Reusable Learning Objectives were highly cost-effective and showed significant effects on outcomes, knowledge, skills, uptake and adherence. Reusable learning Objectives were also highly rated as useful and recommended to others [49]. Studies measuring outcomes during the development of a technical product reported that co-production with users was helpful, and that it provided understanding valuable for the further improvement of the product [55, 59, 67]. In one study the product uncovered unmet health needs among participants [55] and in another study, the final testing revealed that a co-produced product had led to improved symptoms [60]. One study resulted in a non-functioning IT product [51].

There were positive effects of using co-production to improve processes [54, 55, 57, 58, 65], and the combination of quantitative and qualitative measurements led to a greater understanding of the components influencing the results [54, 58, 65] and additional increased engagement by families [54]. The results also specified requirements for further development of tested interventions [58]. D'Young et al. [61] reported that co-production lead to the fulfilment of the effectiveness goals of a service.

Success factors

Close engagement and co-production with those intended to take part in, or benefitting from the intervention, were identified in several *mixed methods studies* as success factors [44, 45, 48]. Other identified success factor was the use of emancipatory process

indicators (for example the use of a emoticon survey on important values) in the implementation and evaluation of projects [56] and facilitator training [45].

Impact on participants

Co-production was an influential experience for participants and organisations [45, 48, 52, 53, 66]. Improved well-being [63], respect and confidence [45], and empowerment [56] were examples of influential experiences. However, participation in co-production projects also lead to paradoxical experiences as wishing to engage more participants, while preferring to work in separate groups: desiring more preparation and discussion, while considering invested time as a concern; and viewing co-design as valuable for improved care, while doubting the realization of co-care in practice [46]. The participation also led to feeling of uncertainty and vulnerability in one study [63]. In their study Revenäs et al. [46] suggested that co-design methods influenced the participants' experiences of the process, thus concluding that such methods need to be adjusted to participating stakeholders and contexts.

Learning outcomes

Co-production led to several unforeseen learning outcomes and learned principles were highly valuable in the next step of improvement and development [56]. One study resulted in learning outcomes such as the importance of motivation; identification of most functional techniques; evaluation of mock-ups with end-users; and the recognition of the influence of informal caregivers [51]. Similar learning outcomes were also presented as results in other studies. Rosso and McGrath [44] summarized the learning outcomes of their study as the importance of: formatting a regional partnership action group; selecting appropriate communities according to needs and preparedness; engaging community champions in each location; establishing a continuous consultation framework relating to each location and the regional action group; co-designing culturally appropriate age-relevant activities in collaboration with local stakeholders; and considering sustainability. Boyd et al. [47] summarized the learning outcomes of their study as the value of using co-design alongside traditional quality improvement methodologies; early engagement with patients; staff buy-in; and individuals trying things outside their comfort zones. Another study brought forward methodological learning outcomes, i.e., the synergy between Lean and Experience-Based Co-Design [50]. Only two studies with a mixed methods design measured sustainability over time [52, 64]. Notable, the only study comparing co-production with other methods, stated that the data gathered (in this case, patient incident reports) was unlikely to have been found through other established methods [68].

Analysis and discussion

General interpretations of results

Knowledge of the outcomes of coproduction is fundamental for assessing the value of co-production projects, and to inform approaches to continually improve services important to citizens. Overall, the studies included in this systematic review showed positive outcomes from co-production but these were difficult to compare across the studies. Co-production was a positive experience for participants and provided learning important to continuous engagement in co-production. Various kinds of measures were used, and the outcomes were presented in a diversity of categorisations. The results of

For peer review only

this systematic review point at three perspectives that are important for measuring the outcomes of co-production. These perspectives, and observations regarding study designs are discussed below.

Perspective 1 – Outputs of co-production processes

The first perspective concerns outputs. Outputs are the results of the processes that are undertaken to contribute to the stated objectives of a project [73]. When outputs are measured continuously, they can also be used to monitor ongoing work [74]. The extensive use of a range of self-developed study-specific measurements in the included studies limited comparisons and cumulative knowledge-building regarding co-production. Two exceptions were found where the validated measures concerned the quality or usability of the intervention itself i.e., outputs of the co-production process. The Mobile Application Rating Scale (MARS) was used to explore users' acceptance and experience of a co-produced App [55] and the System Usability Scale (SUS) was used to assess user experience of a mobile App [51].

A precondition for identifying relevant measures in relation to outputs is that the processes are carefully described, and a fundamental aspect of co-production processes concerns how and when users are involved. In general, these processes have been described in detail; however, the clarity concerning in which phases of the process users participated could have been clearer in many of the studies. Without this, studies appear to have been co-produced even when participation was restricted to only one phase. In their generic program theory for co-production, Brix et al. [18] suggest three phases (co-design/co-planning, co-delivery/co-creation and co-review/co-evaluation) which may be helpful to clarify when and how users participate.

Perspective 2 - Experiences of participating in co-production processes

The second perspective concerns participants' experiences of participating in co-production. Given that co-production is anchored in democratic and equal value principles [11, 13] it is notable that only three studies provided insights into how these core values might be measured. The Relational Coordination Scale (RCS) scale was used to assess how participants perceived the interaction among actors during the collaboration [43] and the National Health and Medical Research Council's (NHMRC) principles guided a strong focus on values important for collaboration with indigenous communities [56]. The self-developed questionnaire by Revenäs et al. [46] measured how participants experienced the possibility of having their voice heard during a co-production collaboration. In general, users' objectives to participate were not well described. As their participation is fundamental for co-production, this is notable. If the initiative takers are unaware of the users' intrinsic and extrinsic aims for participation, they can neither take them into account nor create receptive conditions for them to contribute. There is a need to take users' objectives for participating into explicit account, and to measure experiences of participation.

Perspective 3 - Outcomes of co-production

The third perspective concerns the interventions' stated objectives and expected outcomes [10]. Twenty-six of the 28 validated scales in this systematic review involved this perspective and covered different aspects of improved health or better clinical outcomes. Overall, the studies reported positive outcomes. However, there is a difference between positive outcomes and goal fulfilment. Various kinds of measures

were used but the goal levels for them were seldom set before projects started. Users were seldom involved in the choice of measures or decisions on goal levels. This lack of shared operational objectives makes it difficult to evaluate if the co-production projects reached their expected outcomes – especially from the perspective of users - and has to our knowledge been less problematized in the literature than the lack of shared measurements per se.

Domination of mixed method studies

Mixed method study designs are well-suited for studying complex phenomena such as changes in behaviours or experiences from different perspectives [75]; 60% of the studies in this systematic review used different mixed method study designs. The use of mixed method study design in research in health and social care is increasing [76] but qualitative study designs are typically the most common study designs in co-production research [77]. In qualitative studies, measurements do not have the same central role as in quantitative studies and in the mixed method studies included in this systematic review, qualitative methods were prominent. The study designs identified in this systematic review are in line with both the general trend towards more mixed study designs in health and social care, and the use of qualitative approaches in co-production.

Implications for practice

In this systematic review, we did not identify any generic measures of the outcomes of co-production and nor would we recommend such an approach. Instead, we suggest a model facilitating comparisons between studies and projects that does not limit flexibility and can incorporate the use of both self-developed study-specific measurements and established measurements. In complex contexts, cause-and-effect relationships are not linear and unexpected consequences are common. To address this, a process for selecting a small number of measures representing different perspectives has been suggested [78, 79]. Balanced score card [80] and the value compass [78] are examples of models that seek to help pinpointing different perspectives which are important for measuring and monitoring organisational performance and processes.

We propose a model for balanced measurement of co-production (Figure 3) which combines output measures (Perspective 1) and outcome measures (Perspective 3). Our emphasis on continuous output measurements is in line with a continuous quality improvement approach since this clarify *how* the processes or interventions works. Both perspective 1 and 3 are important to monitor and evaluate change [79]. The model also aligns with the core values of co-production by incorporating how participants experience their own participation in the co-production process (Perspective 2). The model proposes the use of at least one measure per perspective, and the use of both established and self-developed study-specific measures. The chosen measures should be selected to offer actionable information for further improvements.

Figure 3. A model for the balanced measurement of co-production.
Please insert figure 3 here

The notion of "balanced" in this context refers to the act of weighing the outcomes together. A balanced approach may prevent co-production from being used to achieve project objectives without addressing its democratic principles. It may also help focus

For peer review only - http://bmjopen.bmj.com/site/about/guidelines.xhtml

attention on co-production projects in which participants report positive experiences but without the project objectives being fulfilled. Based on how we have found co-production to have been reported in the 43 studies in this systematic review, the model is a radical proposition; only Adinolfi et al. [17] have measures reflecting all three proposed perspectives.

The model also emphasizes the use of both established measures (see online supplemental Appendix 1, first two columns) and self-developed study-specific measures (see online supplemental Appendix 1, third column). Established measures (internationally validated scales and measures based on publications are depicted as a light-grey core at the centre of the model. This core of measurements corresponds with the outcome measures suggested by Marsilio et al. [77] for example: validated scales, other metrics, and indicators. In our systematic review a multitude of measures based on publications were used whereas the use of internationally validated scales was much more restricted. This may indicate a lack of knowledge of existing validated scales. Broadened multidisciplinary competence among researchers and professionals in the field of co-production may support a greater use of validated scales, which could then strengthen the reliability of results and promote comparisons. The model also includes self-developed study-specific measures, placed in the dotted zone surrounding the core (Figure 3). Such measures are "tailor-made" for the specific purposes of a single study and their joint development by users and providers within a study can be a foundation for shared understanding, creativity and engagement.

Our proposal for self-developed study-specific measures is in line with the findings in Greenhalgh et al's systematic review regarding frameworks for patient and public involvement [81], in which they state that it might be more efficient and sustainable to develop one's own framework than to apply someone else's. Another implication from Greenhalgh's et al review relating to our suggested model is the need for the actors themselves to formulate operational objectives, so that they - with their own measures - can determine whether the co-production project has reached its intended goals. Finally, included papers in this systematic review generally showed a low awareness regarding the difference between outputs and outcomes, and we believe that the proposed model can clarify both the differences between the two perspectives, and their shared importance.

Implications for future research

An important implication for research concerns the need for closely described co-production processes, including clarifications of the activities, or process steps, that users participate in. Such clarity would provide better understanding of how co-production activities impact outcomes. A good example of how to make the co-production process in research transparent is provided by Marks et al. [82]. In their scoping review, they list specific citizen science activities and group them into categories describing increasing public involvement (contributory, collaborative, co-created and citizen- lead).

A second implication for future research concerns the composition of project groups, which in general need to include more multidisciplinary competences. A broader knowledge base can facilitate the identification and use of established measures, thus linking local co-production projects to a cumulative body of knowledge. A third

For peer review only

implication concerns the description of measures. In several papers authors refer to measures, surveys or survey questions without describing them. Regardless of the type of measures, these should be described in sufficient detail that researchers can consider them for other studies. A fourth implication concerns the need for better knowledge of which mixed method study designs are most suited for research in co-production in health and welfare services. Lastly, the diversity in how co-production research was reported in the papers included in this systematic review points to a major implication, the development of reporting guidelines for co-production research. The adoption and use of such a guideline would significantly contribute to the scientific maturity of the research field.

Strengths and limitations

This review is in line with the published study protocol [8] and contributes to the aim of exploring, enhancing and measuring the value of co-production for improving the health and social care of citizens. Against the background of the widespread ambitions to integrate health and social care services, whilst the number of empirical studies on co-production is increasing in health care, it is a strength that this systematic review includes both health and social care services. Furthermore, the MMAT tool was crucial for appraising the quality of included studies and provided an overview of the used study designs. The categories used to code and analyse the extracted measures were also easy to use and can be recommended to other studies and projects on co-production. The identified measures are empirically tested which indicates that they are relevant and possible to use in other empirical settings.

The data for this systematic review was obtained from a scoping review, in which only peer review research, published in English were included [7]. Though justified considering the aims of this systematic review, this inherently narrowed our focus and may mean we have missed important applied co-production research published in other languages. We also acknowledge that it would be ideal to update the review. However, the detailed analysis was a time-consuming process and repeating the analysis would create a similar delay between an updated search and publication. Further to this, research exploring Cochrane reviews described that only a minority were updated to incorporate evidence from new primary studies. Of those that did, very few updates led to changes in the conclusions [83]. We believe this applies to our systematic review as well and that the overall findings are most unlikely to change with an update covering only a small number of years The more recently published reviews with which we have compared our results lend weight to this belief.

Conclusions

The objective of this systematic review was to increase understanding of how empirical studies have measured the outcomes of co-production and co-design in health and social care and what the outcomes are. Overall, the studies reported positive outcomes using a multitude of measures. This plurality limited comparisons and cumulative knowledge building but the lack of common strategies and models for measuring co-production is more problematic than the plurality itself. The findings of this systematic review suggest that co-production should be measured from three perspectives: the outputs of co-production processes, the experiences of participating in co-production processes and outcomes of co-production. Two types of measures should be used: self-developed

For peer review only

study-specific measures and established measures. By measuring and monitoring co-production with this balanced approach the democratic aspects of co-production and its complexity can be incorporated within future evaluations. By using the model for the balanced measurement of co-production, we believe that future studies in co-production can better contribute to "the science of participation" [84]. The development of a reporting guideline would also enhance the needed transparency and the scientific maturity of this research field.

Acknowledgments

Authors confirm contribution to the paper were as follows: all authors took part in the study conception and design. AN, DM and KAJ performed the data collection. AN and KAJ performed the data extraction, analysis and interpretation of results. AN and KAJ made the draft manuscript preparation. DM wrote major parts of the method section and AN wrote the discussion section. All authors reviewed the results and approved the final version of the manuscript. We are grateful for the help from all participants in co-production events, from our university library and from colleagues in improving the design of Figure 3. Also, thanks to B. L who improved the design of Figure 3.

Funding

This work is part of the Samskapa program which is supported by Forte: The Swedish Research Council for Health, Working Life and Welfare, grant number 2018-01431.

Abbreviations

MMAT: Mixed Method quality Appraisal Tool
PRISMA: Preferred Reporting Items for Systematic Reviews and Meta-Analyses

Conflicts of interests

The authors declare no conflict of interest.

References

1. Robert G, Donetto S, Williams O. Co-designing healthcare services with patients. The Palgrave Handbook of Co-Production of Public Services and Outcomes: Springer; 2021. p. 313-33.
2. Fusco F, Marsilio M, Guglielmetti C. Co-production in health policy and management: a comprehensive bibliometric review. BMC Health Services Research. 2020;20:1-16. doi: 10.1186/s12913-020-05241-2.
3. Voorberg WH, Bekkers VJ, Tummers LG. A systematic review of co-creation and co-production: Embarking on the social innovation journey. Public Management Review. 2015;17(9):1333-57. doi: 10.1080/14719037.2014.930505.
4. Loeffler E, Bovaird T. User and community co-production of public services: What does the evidence tell us? International Journal of Public Administration. 2016;39(13):1006-19. doi: 10.1080/01900692.2016.1250559.
5. Osborne SP, Radnor Z, Strokosch K. Co-production and the co-creation of value in public services: a suitable case for treatment? Public Management Review. 2016;18(5):639-53. doi: 10.1080/14719037.2015.1111927.

For peer review only

6. Palumbo R. Contextualizing co-production of health care: a systematic literature review. International Journal of Public Sector Management. 2015;29(1):72-90. doi: 10.1108/IJPSM-07-2015-0125
7. Masterson D, Areskoug Josefsson K, Robert G, Elisabeth N, Kjellström S. Mapping definitions of co-production and co-design in health and social care: A systematic scoping review providing lessons for the future. Health Expectations. 2022. doi: 10.1111/hex.13470.
8. Kjellström S, Areskoug-Josefsson K, Gäre BA, Andersson A-C, Ockander M, Käll J, et al. Exploring, measuring and enhancing the coproduction of health and well-being at the national, regional and local levels through comparative case studies in Sweden and England: the 'Samskapa'research programme protocol. BMJ open. 2019;9(7):e029723. doi: 10.1136/bmjopen-2019-029723.
9. Sullivan H, Williams P. Whose kettle?: Exploring the role of objects in managing and mediating the boundaries of integration in health and social care. Journal of Health, Organisation and Management. 2012;26(6):697-712. doi: 10.1108/14777261211276970.
10. Alford J. The multiple facets of co-production: building on the work of Elinor Ostrom. Public Management Review. 2014;16(3):299-316. doi: 10.1080/14719037.2013.806578
11. Holland-Hart DM, Addis SM, Edwards A, Kenkre JE, Wood F. Coproduction and health: Public and clinicians' perceptions of the barriers and facilitators. Health Expectations. 2019;22(1):93-101. doi: 10.1111/hex.12834
12. Verschuere B, Brandsen T, Pestoff V. Co-production: The state of the art in research and the future agenda. Voluntas: International Journal of Voluntary and Nonprofit Organizations. 2012;23(4):1083-101. doi: 10.1007/s11266-012-9307-8
13. Pestoff V. Citizens and co-production of welfare services: Childcare in eight European countries. Public management review. 2006;8(4):503-19. doi: 10.1080/14719030601022882.
14. Baines R, Bradwell H, Edwards K, Stevens S, Prime S, Tredinnick-Rowe J, et al. Meaningful patient and public involvement in digital health innovation, implementation and evaluation: A systematic review. Health Expectations. 2022. doi: 10.1111/hex.13506.
15. Durose C, Needham C, Mangan C, Rees J. Generating'good enough'evidence for co-production. Evidence & Policy: A Journal of Research, Debate and Practice. 2017;13(1):135-51. doi: 10.1332/174426415X14440619792955.
16. Lim S, Morris H, Pizzirani B, Kajewski D, Lee WK, Skouteris H. Evaluating hospital tools and services that were co-produced with patients: A rapid review. International Journal for Quality in Health Care. 2020;32:231-9. doi: 10.1093/intqhc/mzaa020.
17. Adinolfi P, Starace F, Palumbo R. Health outcomes and patient empowerment: The case of health budgets in Italy. Journal of Health Management. 2016;18(1):117-33. doi: 10.1177/0972063415625524
18. Brix J, Krogstrup HK, Mortensen NM. Evaluating the outcomes of co-production in local government. Local Government Studies. 2020;46(2):169-85. doi: 10.1080/03003930.2019.1702530.
19. Lindsay C, Pearson S, Batty E, Cullen AM, Eadson W. Co-production as a route to employability: Lessons from services with lone parents. Public Administration. 2018;96(2):318-32. doi: 10.1111/padm.12408.
20. Greenhalgh T, Jackson C, Shaw S, Janamian T. Achieving research impact through co-creation in community-based health services: literature review and case study. The Milbank Quarterly. 2016;94(2):392-429. doi: 10.1111/1468-0009.12197
21. Filipe A, Renedo A, Marston C. The co-production of what? Knowledge, values, and social relations in health care. PLoS biology. 2017;15(5):e2001403. doi: 10.1371/journal.pbio.2001403.
22. Bombard Y, Baker GR, Orlando E, Fancott C, Bhatia P, Casalino S, et al. Engaging patients to improve quality of care: a systematic review. Implementation Science. 2018;13(1):1-22. doi: 10.1186/s13012-018-0784-z.
23. Clarke D, Jones F, Harris R, Robert G. What outcomes are associated with developing and implementing co-produced interventions in acute healthcare settings? A rapid evidence synthesis. BMJ open. 2017;7(7):e014650. doi: 10.1136/bmjopen-2016-014650

For peer review only

24. Moher D, Liberati A, Tetzlaff J, Altman DG, Group* P. Preferred reporting items for systematic reviews and meta-analyses: the PRISMA statement. Annals of internal medicine. 2009;151(4):264-9. doi: 10.1371/journal.pmed.1000097.
25. Ouzzani M, Hammady H, Fedorowicz Z, Elmagarmid A. Rayyan—a web and mobile app for systematic reviews. Systematic reviews. 2016;5(1):1-10. doi: 10.1186/s13643-016-0384-4
26. Hong QN, Pluye P, Fàbregues S, Bartlett G, Boardman F, Cargo M, et al. Mixed methods appraisal tool (MMAT), version 2018. Registration of copyright. 2018;1148552. doi: 10.3233/EFI-180221.
27. Pluye P, Hong QN. Combining the power of stories and the power of numbers: mixed methods research and mixed studies reviews. Annual review of public health. 2014;35:29-45. doi: 10.1146/annurev-publhealth-032013-182440
28. Areskoug Josefsson K, Nordin A. Insights from co-reflection. Vertikals2020.
29. Denyer D, Tranfield T. Producing a systematic review. In: Buchanan DAaB, A. (Eds), editor. The Sage Handbook of Organizational Research Methods. London: Sage; 2009.
30. Page MJ, McKenzie JE, Bossuyt PM, Boutron I, Hoffmann TC, Mulrow CD, et al. The PRISMA 2020 statement: an updated guideline for reporting systematic reviews. Systematic reviews. 2021;10(1):1-11. doi: 10.1038/nature25753.
31. Sadler E, Porat T, Marshall I, Hoang U, Curcin V, Wolfe CD, et al. Shaping innovations in long-term care for stroke survivors with multimorbidity through stakeholder engagement. PloS one. 2017;12(5). doi: 10.1371/journal.pone.0177102.
32. Stallard P, Porter J, Grist R. A smartphone app (BlueIce) for young people who self-harm: open phase 1 pre-post trial. JMIR mHealth and uHealth. 2018;6(1):e32. doi: 10.2196/mhealth.8917
33. Bucci S, Lewis S, Ainsworth J, Haddock G, Machin M, Berry K, et al. Digital interventions in severe mental health problems: lessons from the Actissist development and trial. World Psychiatry. 2018;17(2):230. doi: 10.1002/wps.20535
34. Hughes-Morley A, Hann M, Fraser C, Meade O, Lovell K, Young B, et al. The impact of advertising patient and public involvement on trial recruitment: embedded cluster randomised recruitment trial. Trials. 2016;17(1):1-13. doi: 10.1186/s13063-016-1718-1.
35. Hastings R, Gillespie D, Flynn S, McNamara R, Taylor Z, Knight R, et al. Who's challenging who training for staff empathy towards adults with challenging behaviour: cluster randomised controlled trial. Journal of intellectual disability research. 2018;62(9):798-813. doi: doi.org/10.1111/jir.12536.
36. Hahn-Goldberg S, Okrainec K, Damba C, Huynh T, Lau D, Maxwell J, et al. Implementing patient-oriented discharge summaries (PODS): a multisite pilot across early adopter hospitals. Healthcare quaterly. 2016;19(1):42-8. doi: 10.12927/hcq.2016.24610
37. Bolton M, Moore I, Ferreira A, Day C, Bolton D. Community organizing and community health: piloting an innovative approach to community engagement applied to an early intervention project in south London. Journal of Public Health. 2016;38(1):115-21. doi: 10.1093/pubmed/fdv017
38. Murphy L, Wells JS, Lachman P, Bergin M. A quality improvement initiative in community mental health in the Republic of Ireland. Health Science Journal. 2015;9(1):1. doi: hdl.handle.net/10147/560881
39. Chapman H, Farndon L, Matthews R, Stephenson J. Okay to Stay? A new plan to help people with long-term conditions remain in their own homes. Primary Health Care Research & Development. 2019;20. doi: 10.1017/S1463423618000786
40. Lamph G, Sampson M, Smith D, Williamson G, Guyers M. Can an interactive e-learning training package improve the understanding of personality disorder within mental health professionals? The Journal of Mental Health Training, Education and Practice. 2018. doi: 10.1108/JMHTEP-03-2017-0023.
41. Turner A, Realpe AX, Wallace LM, Kosmala-Anderson J. A co-produced self-management programme improves psychosocial outcomes for people living with depression. Mental Health Review Journal. 2015;20(4):242-55. doi: 10.1108/MHRJ-05-2014-0017.

For peer review only

42. Roberts CM, Gungor G, Parker M, Craig J, Mountford J. Impact of a patient-specific co-designed COPD care scorecard on COPD care quality: a quasi-experimental study. NPJ primary care respiratory medicine. 2015;25(1):1-5. doi: 10.1038/npjpcrm.2015.17
43. Cramm JM, Nieboer AP. The changing nature of chronic care and coproduction of care between primary care professionals and patients with COPD and their informal caregivers. International journal of chronic obstructive pulmonary disease. 2016;11:175-82. doi: 10.2147/COPD.S94409
44. Rosso E, McGrath R. Promoting physical activity among children and youth in disadvantaged South Australian CALD communities through alternative community sport opportunities. Health Promotion Journal of Australia. 2016;27(2):105-10. doi: doi.org/10.1071/HE15092.
45. Morales-Perez A, Nava-Aguilera E, Legorreta-Soberanis J, Paredes-Solís S, Balanzar-Martínez A, Serrano-de los Santos FR, et al. Which Green Way: description of the intervention for mobilising against Aedes aegypti under difficult security conditions in southern Mexico. BMC Public Health. 2017;17(1):398. doi: 10.1186/s12889-017-4300-1
46. Revenäs Å, Forsberg HH, Granström E, Wannheden C. Co-Designing an eHealth service for the Co-Care of Parkinson disease: explorative study of values and challenges. JMIR research protocols. 2018;7(10):e11278. doi: 10.2196/11278
47. Boyd H, McKernon S, Mullin B, Old A. Improving healthcare through the use of co-design. New Zealand Medical Association. 2012;125(1357):76-87.
48. Petrakis M, Brophy L, Lewis J, Stylianou M, Scott M, Cocks N, et al. Consumer measures and research co-production: A pilot study evaluating the recovery orientation of a mental health program collaboration. Asia Pacific Journal of Social Work and Development. 2014;24(1-2):94-108. doi: 10.1080/02185385.2014.885212.
49. Ferguson M, Maidment D, Henshaw H, Gomez R. Knowledge is power: improving outcomes for patients, partners, and professionals in the digital age. Perspectives of the ASHA Special Interest Groups. 2019;4(1):140-8. doi: 10.1044/2018_PERS-SIG7-2018-0006.
50. Brosseau M, Gagnon D, Rohde K, Schellinck J. Integrating Engagement and Improvement Work in a Pediatric Hospital. Healthcare quarterly 2017;20(1):67-72. doi: 10.12927/hcq.2017.25071
51. Garzo A, Silva PA, Garay-Vitoria N, Hernandez E, Cullen S, De Cock VC, et al. Design and development of a gait training system for Parkinson's disease. PloS one. 2018;13(11). doi: 10.1371/journal.pone.0207136.
52. Davies J, Sampson M, Beesley F, Smith D, Baldwin V. An evaluation of Knowledge and Understanding Framework personality disorder awareness training: Can a co-production model be effective in a local NHS mental health Trust? Personality and mental health. 2014;8(2):161-8. doi: 10.1002/pmh.1257
53. Montenegro M, Greenhill B. Evaluating 'freda challenge': a coproduced human rights board game in services for people with intellectual disabilities. Journal of Applied Research in Intellectual Disabilities. 2015;28(3):223-37. doi: 10.1111/jar.12124
54. Khan A, Spector ND, Baird JD, Ashland M, Starmer AJ, Rosenbluth G, et al. Patient safety after implementation of a coproduced family centered communication programme: multicenter before and after intervention study. BMJ. 2018;363. doi: 10.1136/bmj.k4764.
55. Webb MJ, Wadley G, Sanci LA. Improving patient-centered care for young people in general practice with a codesigned screening app: mixed methods study. JMIR mHealth and uHealth. 2017;5(8):e118. doi: 10.2196/mhealth.7816
56. Haynes E, Marawili M, Marika BM, Mitchell AG, Phillips J, Bessarab D, et al. Community-based participatory action research on rheumatic heart disease in an Australian Aboriginal homeland: Evaluation of the 'On track watch'project. Evaluation and program planning. 2019;74:38-53. doi: 10.1016/j.evalprogplan.2019.02.010.
57. Rawson TM, Moore LS, Castro-Sanchez E, Charani E, Hernandez B, Alividza V, et al. Development of a patient-centred intervention to improve knowledge and understanding of antibiotic therapy in secondary care. Antimicrobial Resistance & Infection Control. 2018;7(1):1-10. doi: 10.1186/s13756-018-0333-1

For peer review only

58. Tsianakas V, Robert G, Richardson A, Verity R, Oakley C, Murrells T, et al. Enhancing the experience of carers in the chemotherapy outpatient setting: an exploratory randomised controlled trial to test impact, acceptability and feasibility of a complex intervention co-designed by carers and staff. Supportive Care Cancer. 2015;23(10):3069-80. doi: 10.1007/s00520-015-2677-x
59. Revenäs Å, Opava CH, Ahlén H, Brusewitz M, Pettersson S, Åsenlöf P. Mobile internet service for self-management of physical activity in people with rheumatoid arthritis: evaluation of a test version. RMD open. 2016;2(1):e000214. doi: 10.1136/rmdopen-2015-000214
60. Baxter S, Reed H, Clarke Z, Judge S, Heron N, Mccarthy A, et al. Evaluating a novel cervical orthosis, the Sheffield Support Snood, in patients with amyotrophic lateral sclerosis/motor neuron disease with neck weakness. Amyotrophic Lateral Sclerosis and Frontotemporal Degeneration. 2016;17(5-6):436-42. doi: 10.3109/21678421.2016.1148170
61. d'Young AI, Young L, Ockelford PA, Brasser M, Slavin K, Manson L, et al. The use of a co-design model in improving timely bleed reporting by adults with haemophilia living in the Auckland region of New Zealand. Clinical Haemophilia. 2014;20(3):388-97. doi: 10.1111/hae.12336
62. Eades S. Impact evaluation of an Independent Mental Health Advocacy (IMHA) service in a high secure hospital: a co-produced survey measuring self-reported changes to patient self-determination. Mental health and social inclusion. 2018. doi: 10.1108/MHSI-12-2017-0054
63. Gillard S, Foster R, Turner K. Evaluating the prosper peer-led peer support network: A participatory, coproduced evaluation. Mental Health and Social Inclusion. 2016. doi: 10.1108/MHSI-12-2015-0045
64. Henshall C, Taylor B, Goodwin L, Farre A, Jones ME, Kenyon S. Improving the quality and content of midwives' discussions with low-risk women about their options for place of birth: Co-production and evaluation of an intervention package. Midwifery. 2018;59:118-26. doi: 10.1016/j.midw.2018.01.016
65. Lucock M, Bartys S, Cupac J, Delgadillo J, Denton C, Gaines S, et al. Using Implementation Intentions to Prevent Relapse after Psychological Treatment for Depression–the SMArT Intervention. Behavioural and cognitive psychotherapy. 2018;46(5):626-32. doi: 10.1017/S1352465818000255
66. Taylor A, Dorer G, Gleeson K. Evaluation of a peer support specialist led group. Mental Health and Social Inclusion. 2018. doi: 10.1108/MHSI-03-2018-0012
67. Ogrin R, Viswanathan R, Aylen T, Wallace F, Scott J, Kumar D. Co-design of an evidence-based health education diabetes foot app to prevent serious foot complications: a feasibility study. Practical Diabetes. 2018;35(6):203-9d. doi: 10.1002/pdi.2197
68. Armitage G, Moore S, Reynolds C, Laloë P-A, Coulson C, McEachan R, et al. Patient-reported safety incidents as a new source of patient safety data: an exploratory comparative study in an acute hospital in England. Journal of health services research & policy. 2018;23(1):36-43. doi: 10.1177/1355819617727563
69. Sundram F, Hawken SJ, Stasiak K, Lucassen MF, Fleming T, Shepherd M, et al. Tips and traps: lessons from codesigning a clinician e-monitoring tool for computerized cognitive behavioral therapy. JMIR mental health. 2017;4(1):e3. doi: 10.2196/mental.5878
70. Farmer J, Carlisle K, Dickson-Swift V, Teasdale S, Kenny A, Taylor J, et al. Applying social innovation theory to examine how community co-designed health services develop: using a case study approach and mixed methods. BMC health services research. 2018;18(1):68. doi: 10.1186/s12913-018-2852-0
71. Litchfield IJ, Bentham LM, Lilford RJ, McManus RJ, Hill A, Greenfield S. Adaption, implementation and evaluation of collaborative service improvements in the testing and result communication process in primary care from patient and staff perspectives: a qualitative study. BMC health services research. 2017;17(1):615. doi: 10.1186/s12913-017-2566-8
72. Hackett CL, Mulvale G, Miatello A. Co-designing for quality: Creating a user-driven tool to improve quality in youth mental health services. Health Expectations. 2018;21(6):1013-23. doi: 10.1111/hex.12694
73. Levine D, Duszak R, Jr. Evolving Priorities for Imaging Utilization Research: From Outputs to Outcomes. JAMA. 2019;322(9):819-21. doi: 10.1001/jama.2019.10158.

For peer review only

74. Nelson EC, Batalden PB, Godfrey MM. Measurment and monitoring. In: Nelson EC, Batalden PB, Godfrey MM, editors. Quality by Design - a clinical microsystem approach: John: Bass; 2007.
75. Morse JM. Mixed method design: Principles and procedures: Routledge; 2016. ISBN: 1315424525.
76. Curry LA, Krumholz HM, O'Cathain A, Clark VLP, Cherlin E, Bradley EH. Mixed methods in biomedical and health services research. Circulation: Cardiovascular Quality and Outcomes. 2013;6(1):119-23. doi: 10.1161/CIRCOUTCOMES.112.967885
77. Marsilio M, Fusco F, Gheduzzi E, Guglielmetti C. Co-Production Performance Evaluation in Healthcare. A Systematic Review of Methods, Tools and Metrics. International journal of environmental research and public health. 2021;18(7):3336. doi: 10.3390/ijerph18073336

78. Nelson EC, Batalden PB, Godfrey MM. Quality by design: a clinical microsystems approach. San Fransisco: Jossey: Bass; 2007. ISBN: 1118046838.
79. Langley GJ, Moen R, Nolan KM, Nolan TW, Norman CL, Provost LP. The improvement guide: a practical approach to enhancing organizational performance. 2nd ed. San Fransisco: Jossey-Bass; 2009. ISBN: 9780470192412.
80. Kaplan RS, Norton DP. Linking the balanced scorecard to strategy. California management review. 1996;39(1):53-79. doi: 10.2307/41165876.
81. Greenhalgh T, Hinton L, Finlay T, Macfarlane A, Fahy N, Clyde B, et al. Frameworks for supporting patient and public involvement in research: systematic review and co-design pilot. Health expectations. 2019;22(4):785-801. doi: 10.1111/hex.12888.
82. Marks L, Laird Y, Trevena H, Smith BJ, Rowbotham S. A Scoping Review of Citizen Science Approaches in Chronic Disease Prevention. Frontiers in Public Health. 2022:953. doi: 10.3389/fpubh.2022.743348.
83. Bashir R, Surian D, Dunn AG. Time-to-update of systematic reviews relative to the availability of new evidence. Systematic Reviews. 2018;7(1):1-8. doi: 10.1186/s13643-018-0856-9.
84. Palmer VJ. The participatory zeitgeist in health care: it is time for a science of participation. Journal of Participatory Medicine. 2020;12(1):e15101.

For peer review only

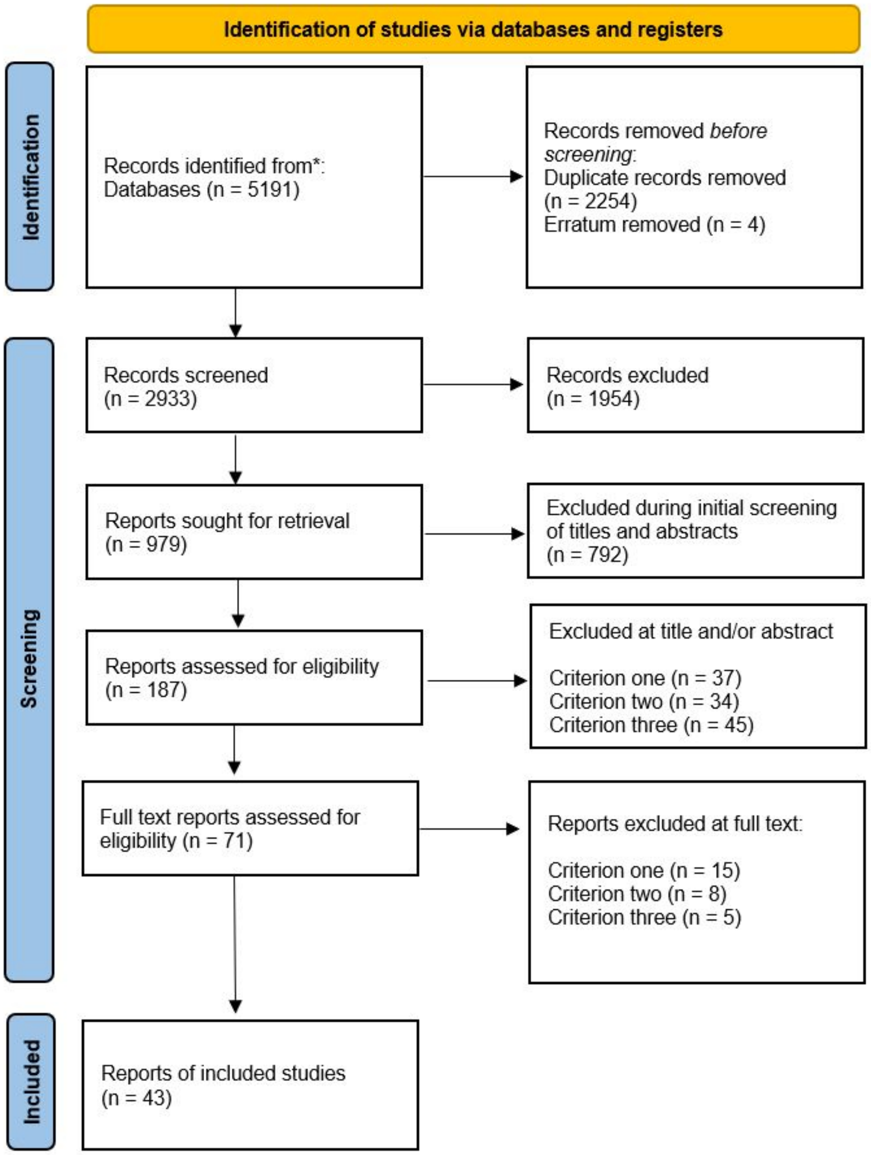

The PRISMA flow diagram

144x187mm (125 x 125 DPI)

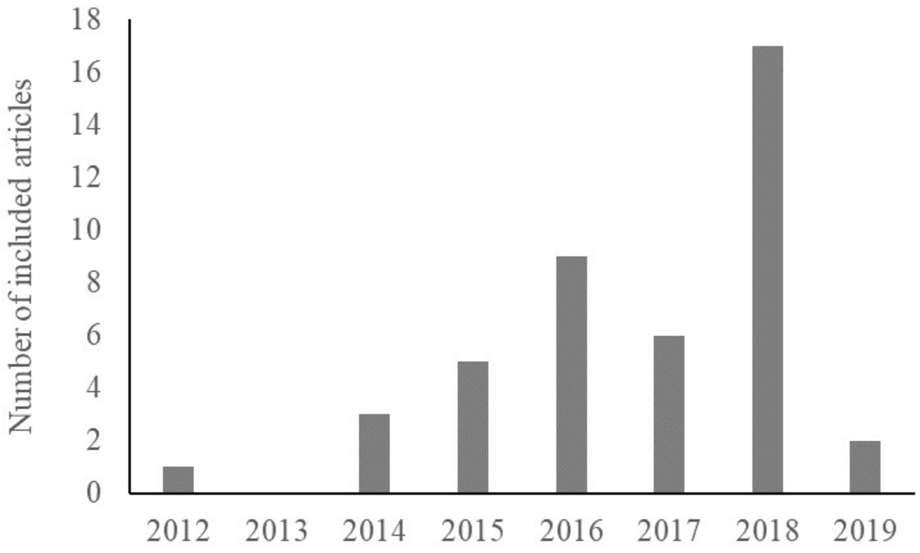
Number of included articles
0
2
4
6
8
10
12
14
16
18
2012
2013
2014
2015
2016
2017
2018
2019

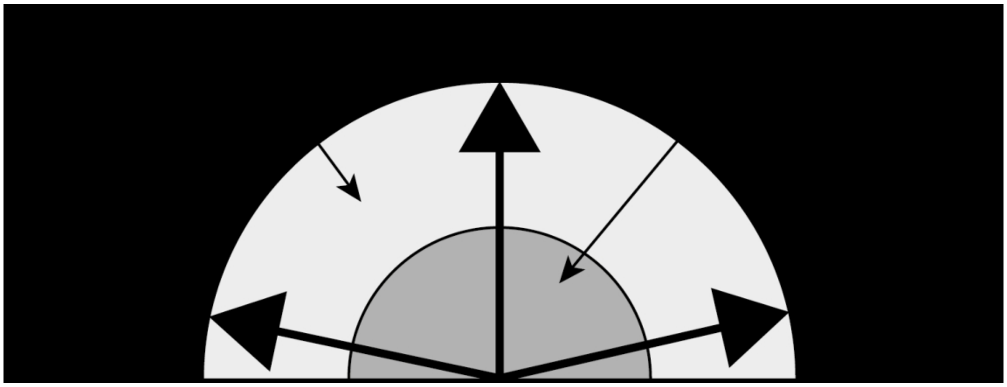

A model for the balanced measurement of co-production

128x48mm (300 x 300 DPI)

The measurement and outcomes of co-production in health and social care – a systematic review of empirical studies

Supplementary file 1. Search strategy for each database

**Search terms to be included in title, abstract or key words**
(Co-produc* OR coproduc* OR co-design* OR codesign*)<br>**AND** (health OR social OR "Public Service*" OR “public sector”)<br>**AND NOT** (engineer* OR biomass OR bioproduct* OR osmosis OR microalgae OR ecosystems OR hydrogen OR Brand OR Algebra)

Note: At the time of our search (March 2019) and writing, the searched databases lacked controlled vocabulary or subject headings for ‘co-production’, so the decision was made to only search in the title, abstract, and key word (subjects) fields. Excluding irrelevant contexts was aided through the search strategy (engineer* OR biomass OR bioproduct* OR osmosis OR microalgae OR ecosystems OR hydrogen OR Brand OR Algebra). An alternative to incorporating these terms could have been to only search subject specific databases (e.g., MEDLINE) but this may have omitted a significant number of relevant papers.

CINAHL with Full Text (EBSCOHost) – 588 hits on March 18, 2019
TI ((co-produc* OR coproduc* OR co-design* OR codesign*) AND (health OR social OR “public service*” OR “public sector*”) NOT (engineer* OR biomass OR chemic* OR bioproduct* OR osmosis OR microalgae OR ecosystems OR hydrogen OR brand OR algebra)) OR AB ((co-produc* OR coproduc* OR co-design* OR codesign*) AND (health OR social OR “public service*” OR “public sector*”) NOT (engineer* OR biomass OR chemic* OR bioproduct* OR osmosis OR microalgae OR ecosystems OR hydrogen OR brand OR algebra))
Limiters - Peer Reviewed; English Language

Cochrane Central Register of Controlled Trials (Wiley) – 47 hits on March 19, 2019
(co-produc* OR coproduc* OR co-design* OR codesign*):ti,ab,kw AND (health OR social OR “public service*” OR “public sector*”):ti,ab,kw NOT (engineer* OR biomass OR chemic* OR bioproduct* OR osmosis OR microalgae OR ecosystems OR hydrogen OR brand OR algebra):ti,ab,kw

MEDLINE (EBSCOHost) – 793 hits on March 18, 2019
TI ((co-produc* OR coproduc* OR co-design* OR codesign*) AND (health OR social OR “public service*” OR “public sector*”) NOT (engineer* OR biomass OR chemic* OR bioproduct* OR osmosis OR microalgae OR ecosystems OR hydrogen OR brand OR algebra)) OR AB ((co-produc* OR coproduc* OR co-design* OR codesign*) AND (health OR social OR “public service*” OR “public sector*”) NOT (engineer* OR biomass OR chemic* OR bioproduct* OR osmosis OR microalgae OR ecosystems OR hydrogen OR brand OR algebra))

For peer review only

Limiters – English Language

PsycINFO (ProQuest) – 403 hits on March 18, 2019
ti((co-produc* OR coproduc* OR co-design* OR codesign*) AND (health OR social OR "public service*" OR "public sector*") NOT (engineer* OR biomass OR chemic* OR bioproduct* OR osmosis OR microalgae OR ecosystems OR hydrogen OR brand OR algebra)) OR ab((co-produc* OR coproduc* OR co-design* OR codesign*) AND (health OR social OR "public service*" OR "public sector*") NOT (engineer* OR biomass OR chemic* OR bioproduct* OR osmosis OR microalgae OR ecosystems OR hydrogen OR brand OR algebra))
Limits – Peer reviewed; Language: English

PubMed (legacy) – 898 hits on March 18, 2019
Search (((co-produc*[Title/Abstract] OR coproduc*[Title/Abstract] OR co-design*[Title/Abstract] OR codesign*[Title/Abstract])) AND (health[Title/Abstract] OR social[Title/Abstract] OR “public service*”[Title/Abstract] OR “public sector*”[Title/Abstract])) NOT (engineer*[Title/Abstract] OR biomass[Title/Abstract] OR chemic*[Title/Abstract] OR bioproduct*[Title/Abstract] OR osmosis[Title/Abstract] OR microalgae[Title/Abstract] OR ecosystems[Title/Abstract] OR hydrogen[Title/Abstract] OR brand[Title/Abstract] OR algebra[Title/Abstract])
Filters: English

Scopus (Elsevier) – 2462 hits on March 18, 2019
(TITLE-ABS (co-produc* OR coproduc* OR co-design* OR codesign*) AND TITLE-ABS (health OR social OR "public service*" OR "public sector*") AND NOT TITLE-ABS (engineer* OR biomass OR chemic* OR bioproduct* OR osmosis OR microalgae OR ecosystems OR hydrogen OR brand OR algebra)) AND (LIMIT-TO (LANGUAGE , "English"))

For peer review only

Appendix 1. Studies, outcomes and measures.

Qualitative studies				
**Paper**	**International validated scales**	**Measures based on publications**	**Self-developed study-specific measures**	**Measures on the experience of co-production**
Tips and Traps: Lessons From Co-designing a Clinician E-Monitoring Tool for Computerized Cognitive Behavioral Therapy [69]				
Shaping innovations in long-term care for stroke survivors with multimorbidity through stakeholder engagement [31]				
Adaption, implementation and evaluation of collaborative service improvements in the testing and result communication process in primary care from patient and staff perspectives: a qualitative study [71]				
Applying social innovation theory to examine how community co-designed health services develop: using a case study approach and mixed methods [70]				
Co-designing for quality: Creating a user-driven tool to improve quality in youth mental health services [72]				

Note Empty fields in the table indicate that no measures were identified. In the qualitative studies, measures are not applicable.

For peer review only

Quantitative randomized control trials				
**Papers**	**International validated scales**	**Measures based on publications**	**Self-developed study-specific measures**	**Measures on the experience of co-production**
A Smartphone App (BlueIce) for Young People Who Self-Harm: Open Phase 1 Pre-Post Trial [32]	Strengths and Difficulties Questionnaire (SDQ)<br><br>Mood and Feelings Questionnaire (MFQ)<br><br>Revised Child Anxiety and Depression Scale (RCADS)		The intervention and self-harm	
Digital interventions in severe mental health problems: Lessons from the Actissist development and trial [33]	Positive and Negative Syndrome Scale (PANSS)<br><br>Calgary Depression Scale for Schizophrenia (CDSS)		The intervention and safety	
Who's challenging who training for staff empathy towards adults with challenging behaviour: cluster randomised controlled trial [35]	Maslach Burnout Inventory (MBI)<br><br>Community Living Attitudes Scale (CLAS-MR)<br><br>Staff Positive Contributions Questionnaire (SPCQ)	Staff Empathy for people with Challenging Behavior Questionnaire (SECBQ)		
The impact of advertising patient and public involvement on trial recruitment: embedded cluster randomized recruitment trial [34]			Effects of the intervention	

For peer review only

Quantitative non-randomized controlled studies				
**Papers**	**International validated scales**	**Measures based on publications**	**Self-developed study-specific measures**	**Measures on the experience of co-production**
Implementing Patient-Oriented Discharge Summaries (PODS): A Multisite Pilot Across Early Adopter Hospitals [36]			Implementation process and intervention functionality	
A quality improvement initiative in community mental health in the republic of Ireland [38]			Experiences of the improvements	
Community organizing and community health: piloting an innovative approach to community engagement applied to an early intervention project in south London [37]	The General Health Questionnaire-12 (GHQ-12)<br><br>The Warwick-Edinburgh Mental Well-being Scale (WEMWBS)	Social Support Program Acceptability Rating Scale (The Treatment Acceptability Rating Scale)<br><br>Functionality of the project (NICE)<br><br>The Adapted Social Capital Questionnaire (World Bank's Social Capital Integrated Questionnaire)	Activities in the program	

For peer review only

Quantitative descriptive studies				
**Papers**	**International validated scales**	**Measures based on publications**	**Self-developed study-specific measures**	**Measures on the experience of co-production**
A co-produced self-management programme improves psychosocial outcomes for people living with depression [41]	Patient Activation Measure (PAM)<br><br>Patient Health Questionnaire (PHQ-9)<br><br>Hospital Anxiety Depression Scale (HADS)<br><br>EuroQol<br><br>Health Education Impact Questionnaire (heiQ)			
Impact of a patient-specific co-designed COPD care scorecard on COPD care quality: a quasi-experimental study [42]		Six quality indicators (NICE) matched against data from clinical records	Clinical outcomes of the change	
Health Outcomes and Patient Empowerment: The case of Health budgets in Italy [17]	The Health of the Nation Outcome Scale, Italian version (HoNOS)	Levels of co-production intensity	Costs of a program	
The changing nature of chronic care and co-production of care between primary care professionals and patients with COPD and their informal caregivers [43]	Patient Assessment of Chronic Illness Care questionnaire (PACIC)<br><br>Assessment of Chronic Illness Care, Short version (ACIC-S)			RCS to assess perceptions of interaction productivity
Can an interactive e-learning training package improve the understanding of personality disorder within mental health professionals? [40]		The Personality Disorder-Knowledge, Attitudes and Skills Questionnaire (PD-KASQ)		

For peer review only

		Efficiency of intervention (researchers' earlier study)		
Okay to Stay? A new plan to help people with long-term conditions remain in their own homes [39]			Efficiency of the program	

For peer review only

Mixed method studies				
**Papers**	**International validated scales**	**Measures based on publications**	**Self-developed study-specific measures**	**Measures on the experience of co-production**
Improving healthcare through the use of co-design [47]		Experiences of program and number of ideas. (NHS survey)		
An evaluation of Knowledge and Understanding Framework Personality Disorder Awareness Training: Can a co-production model be effective in a local NHS mental health Trust? [52]		The Personality Disorder-Knowledge, Attitudes and Skills Questionnaire (PD-KASQ)	Effects of the program	
Consumer measures and research co-production: A pilot study evaluating the recovery orientation of a mental health program collaboration [48]	The Recovery Self-Assessment (RSA)<br><br>The Recovery Enhancing Environment measure (REE)			
The use of a co-design model in improving timely bleed reporting by adults with haemophilia living in the Auckland region of New Zealand [61]		Mean bleed reporting time<br><br>Bleeds per month<br><br>Bleeding episodes outside of a prespecified 48-h	The clinical effects of the program	
Evaluating 'FREDA Challenge': A co-produced human rights board game in services for people with intellectual disabilities [53]		Human right quiz<br><br>Attitudes to Human Rights questionnaire (ATHIRI)	The attitudes, knowledge, and opinions of the intervention	
Evaluating a novel cervical orthosis, the Sheffield Support Snood, in patients with amyotrophic lateral sclerosis/motor neuron disease with neck weakness [60]			The experiences, comfort and satisfaction with the intervention	
Evaluating the Prosper peer-led peer support network: A participatory, coproduced evaluation [63]			The experiences and priorities on the service	

For peer review only

Mobile internet service for self-management of physical activity in people with rheumatoid arthritis: evaluation of a test version [59]			The innovation, self-monitoring and feedback	
Promoting physical activity among children and youth in disadvantaged South Australian CALD communities through alternative community sport opportunities [44]			The thoughts and feelings of the project	
Enhancing the experience of carers in the chemotherapy outpatient setting: an exploratory randomised controlled trial to test impact, acceptability and feasibility of a complex intervention co-designed by carers and staff [58]	The General Health Questionnaire-12 (GHQ-12)<br><br>Supportive Care Needs Survey for Partners and Carers [SCNS-P&C)	Perceived confidence in supporting friend or relative	The effects of the intervention; knowledge and experiences of care	
Improving Patient-Centered Care for Young People in General Practice With a Co-designed Screening App: Mixed Methods Study [55]	Mobile Application Rating Scale (MARS)	Patient-centered care<br><br>Disclosure<br>Fear of judgment<br>App acceptability<br><br>Knowledge, confidence and enthusiasm<br><br>Youth friendly care	The opinions and willingness to use the innovation	
Integrating Engagement and Improvement Work In a Paediatric Hospital [50]			Impressions and feelings about integrating EBCD and quality improvement tools	
Which green way: description of the intervention for mobilising against Aedes aegypti under difficult security conditions in southern Mexico[45]		Clinical indicators<br><br>Proved infections	Self-reported health outcomes	
Impact evaluation of an Independent Mental Health Advocacy (IMHA) service in a high secure hospital: a co-produced survey measuring self-reported changes to patient self-determination [62]			The improved services' relevance, accessibility and ease of use	

For peer review only

Improving the quality and content of midwives' discussions with low-risk women about their options for place of birth: Co-production and evaluation of an intervention package [64]			The level of knowledge and confidence regarding safety and intervention rates, views of the intervention package<br><br>Objective knowledge calculated using multiple-choice questions	
Design and development of a gait training system for Parkinson's disease [51]	System Usability Scale (SUS)		The development and improvement of a customer focused innovation	
Patient safety after implementation of a co-produced family centered communication programme: multicenter before and after intervention study [54]		Medical errors<br><br>Harmful errors<br><br>Adverse events	Family experiences	
Co-design of an evidence-based health education diabetes foot app to prevent serious foot complications: a feasibility study [67]	The Nottingham Assessment of Functional Footcare (NAFF)	Diabetes foot care knowledge<br><br>Attitudes		
Evaluation of a peer support specialist led group [66]			The experiences and effects of the recovery group<br><br>The number of referrals and contacts	
Patient-reported safety incidents as a new source of patient safety data: an exploratory comparative study in an acute hospital in England [68]			The number of safety incidents counted and compared by methods	
Using Implementation Intentions to Prevent Relapse after Psychological Treatment for Depression - the SMArT Intervention [65]	Patient Health Questionnaire (PHQ-9)<br><br>Generalized Anxiety Disorder Questionnaire (GAD-7)		Counts of self-regulatory strategies, IMPS	

For peer review only

Development of a patient-centred intervention to improve knowledge and understanding of antibiotic therapy in secondary care [57]		Patient knowledge and understanding surrounding antimicrobial therapy and resistance		
Co-Designing an eHealth Service for the Co-Care of Parkinson Disease: Explorative Study of Values and Challenges [46]			The experiences and views of the workshops and logistics	Experience: Questionnaire on participants' overall experiences of participation
Knowledge Is Power: Improving Outcomes for Patients, Partners, and Professionals in the Digital Age [49]	Glasgow Hearing Aid Benefit Profile (GHABP)<br><br>The International Outcome Inventory for Hearing Aids (IOI-HA)<br><br>The Hearing Handicap Inventory for the Elderly (HHIE)		Patient feedback on how they experienced the innovation	
Community-based participatory action research on rheumatic heart disease in an Australian Aboriginal homeland: Evaluation of the 'On track watch' project [56]		Values and Ethics Guidelines, National Health & Medical Research Council<br><br>'How-to' guide, National Health Medical Research Council		Emoticon survey based on National Health and Medical Research Council principles<br>Reciprocity, respect<br>Equality<br>Responsibility<br>Survival and Protection<br>Spirit & integrity

For peer review only

PRISMA 2020 Checklist for: The measurement and outcomes of co-production on health and social care – a systematic review of empirical studies

Section and Topic	Item #	Checklist item	Location where item is reported
**TITLE**			
Title	1	Identify the report as a systematic review.	Title
**ABSTRACT**			
Abstract	2	See the PRISMA 2020 for Abstracts checklist.	Abstract
**INTRODUCTION**			
Rationale	3	Describe the rationale for the review in the context of existing knowledge.	Introduction Sections starting with "There are several plausible explanations related" and "There are a small number of reviews…"
Objectives	4	Provide an explicit statement of the objective(s) or question(s) the review addresses.	Last section of the introduction.
**METHODS**			
Eligibility criteria	5	Specify the inclusion and exclusion criteria for the review and how studies were grouped for the syntheses.	The subsections Information sources, eligibility criteria, search strategy and selection process for the scoping review and Eligibility criteria and selection process for this systematic review.
Information sources	6	Specify all databases, registers, websites, organisations, reference lists and other sources searched or consulted to identify studies. Specify the date when each source was last searched or consulted.	Supplementary file 1.
Search strategy	7	Present the full search strategies for all databases, registers and websites, including any filters and limits used.	Supplementary file 1.
Selection process	8	Specify the methods used to decide whether a study met the inclusion criteria of the review, including how many reviewers screened each record and each report retrieved, whether they worked independently, and if applicable, details of automation tools used in the process.	The subsections Information sources, eligibility criteria, search strategy and selection process for the scoping review and Eligibility criteria and selection process for this systematic review.
Data collection process	9	Specify the methods used to collect data from reports, including how many reviewers collected data from each report, whether they worked independently, any processes for obtaining or confirming data from study investigators, and if applicable, details of automation tools used in the process.	Subsections Eligibility criteria and selection process for this systematic review.
Data items	10a	List and define all outcomes for which data were sought. Specify whether all results that were compatible with each outcome domain in each study were sought (e.g., for all measures, time points, analyses), and if not, the methods used to decide which results to collect.	Table 1 and Synthesis methods.
	10b	List and define all other variables for which data were sought (e.g., participant and intervention characteristics, funding sources). Describe any assumptions made about any missing or unclear information.	Table 2 and Synthesis methods.
Study risk of bias assessment	11	Specify the methods used to assess risk of bias in the included studies, including details of the tool(s) used, how many reviewers assessed each study and whether they worked independently, and if applicable, details of automation tools used in the process.	Subsections Eligibility criteria and selection process for this systematic review.
Effect measures	12	Specify for each outcome the effect measure(s) (e.g., risk ratio, mean difference) used in the synthesis or presentation of results.	N/A

For peer review only

PRISMA 2020 Checklist for: The measurement and outcomes of co-production on health and social care – a systematic review of empirical studies

Section and Topic	Item #	Checklist item	Location where item is reported
Synthesis methods	13a	Describe the processes used to decide which studies were eligible for each synthesis (e.g., tabulating the study intervention characteristics and comparing against the planned groups for each synthesis (item #5)).	Subsections Data collection process ("To develop a shared work procedure…") and Data items for this systematic review.
	13b	Describe any methods required to prepare the data for presentation or synthesis, such as handling of missing summary statistics, or data conversions.	Subsections Data collection process and data items for this systematic review and Synthesis methods.
	13c	Describe any methods used to tabulate or visually display results of individual studies and syntheses.	Table 3.
	13d	Describe any methods used to synthesize results and provide a rationale for the choice(s). If meta-analysis was performed, describe the model(s), method(s) to identify the presence and extent of statistical heterogeneity, and software package(s) used.	Subsection Synthesis methods.
	13e	Describe any methods used to explore possible causes of heterogeneity among study results (e.g., subgroup analysis, meta-regression).	N/A
	13f	Describe any sensitivity analyses conducted to assess robustness of the synthesized results.	N/A
Reporting bias assessment	14	Describe any methods used to assess risk of bias due to missing results in a synthesis (arising from reporting biases).	Subsection Strengths and limitations.
Certainty assessment	15	Describe any methods used to assess certainty (or confidence) in the body of evidence for an outcome.	N/A
**RESULTS**			
Study selection	16a	Describe the results of the search and selection process, from the number of records identified in the search to the number of studies included in the review, ideally using a flow diagram.	Subsection Study selection and Figure 1.
	16b	Cite studies that might appear to meet the inclusion criteria, but which were excluded, and explain why they were excluded.	N/A
Study characteristics	17	Cite each included study and present its characteristics.	Subsection Study characteristics and Appendix 1.
Risk of bias in studies	18	Present assessments of risk of bias for each included study.	N/A
Results of individual studies	19	For all outcomes, present, for each study: (a) summary statistics for each group (where appropriate) and (b) an effect estimate and its precision (e.g. confidence/credible interval), ideally using structured tables or plots.	N/A
Results of syntheses	20a	For each synthesis, briefly summarise the characteristics and risk of bias among contributing studies.	N/A
	20b	Present results of all statistical syntheses conducted. If meta-analysis was done, present for each the summary estimate and its precision (e.g., confidence/credible interval) and measures of statistical heterogeneity. If comparing groups, describe the direction of the effect.	N/A
	20c	Present results of all investigations of possible causes of heterogeneity among study results.	N/A
	20d	Present results of all sensitivity analyses conducted to assess the robustness of the synthesized results.	N/A
Reporting biases	21	Present assessments of risk of bias due to missing results (arising from reporting biases) for each synthesis assessed.	N/A
Certainty of evidence	22	Present assessments of certainty (or confidence) in the body of evidence for each outcome assessed.	N/A
**DISCUSSION**			
Discussion	23a	Provide a general interpretation of the results in the context of other evidence.	Subsection General interpretation of results.
	23b	Discuss any limitations of the evidence included in the review.	Subsection Strengths and limitations.
	23c	Discuss any limitations of the review processes used.	Subsection Strengths and limitations.

For peer review only

PRISMA 2020 Checklist for: The measurement and outcomes of co-production on health and social care – a systematic review of empirical studies

Section and Topic	Item #	Checklist item	Location where item is reported
	23d	Discuss implications of the results for practice, policy, and future research.	Subsections Implications for practice and Implications for future research.
**OTHER INFORMATION**			
Registration and protocol	24a	Provide registration information for the review, including register name and registration number, or state that the review was not registered.	Introduction. Section starting "In the published Samskapa study protocol…"
	24b	Indicate where the review protocol can be accessed, or state that a protocol was not prepared.	Introduction.
	24c	Describe and explain any amendments to information provided at registration or in the protocol.	Subsection Strengths and limitations.
Support	25	Describe sources of financial or non-financial support for the review, and the role of the funders or sponsors in the review.	Subsection Funding.
Competing interests	26	Declare any competing interests of review authors.	Subsection Conflict of interest.
Availability of data, code and other materials	27	Report which of the following are publicly available and where they can be found: template data collection forms; data extracted from included studies; data used for all analyses; analytic code; any other materials used in the review.	None*

'* Research Data at the School of Health and Welfare is regulated under the Freedom of the Press Act (1949:105) and the Public Access to Information and Security Act (2009:4) as Public Records/Official Documents. Research data that is registered and Archived at the School of Health and Welfare can be requested anonymously by anyone for a fixed fee according to the Fee Regulation (1992:191). If the research data contain sensitive information, like personal data and/or trade secrets, that information is protected by Confidentiality and Secrecy by the Public Access to Information and Security Act and requests of such information will be denied with the possibility to appeal the denial in the Court of Appeals. Secret or Confidential research data can be accessed by other researchers if they receive permission from the Regional Ethics Review Board. The lawful basis for transmission of Secret and/or Confidential information is then based on the Law of Ethics Review for Research on Humans (2003:460)

For peer review only

BMJ Open

The measurement and outcomes of co-production in health and social care - a systematic review of empirical studies

Journal:	*BMJ Open*
Manuscript ID	bmjopen-2023-073808.R1
Article Type:	Original research
Date Submitted by the Author:	07-Jul-2023
Complete List of Authors:	Nordin, Annika; Jönköping University School of Health and Welfare, Jönköping Academy of Improvement of Health and Welfare<br>Kjellstrom, Sofia; Jönköping University School of Health and Welfare, Jönkoping Academy of Improvement of Health and Welfare<br>Robert, Glenn; King's College London, Florence Nightingale Faculty of Nursing, Midwifery & Palliative Care,<br>Masterson, Daniel; Jönköping University School of Health and Welfare, Jönköping Academy for Improvement of Health and Welfare<br>Areskoug Josefsson, Kristina; University West, Department of Health Sciences; Jönköping University School of Health and Welfare, Jönköping Academy of Improvement of Health and Welfare
**Primary Subject Heading**:	Health services research
Secondary Subject Heading:	Patient-centred medicine
Keywords:	Patient Participation, Quality in health care < HEALTH SERVICES ADMINISTRATION & MANAGEMENT, Systematic Review

SCHOLARONE™
Manuscripts

I, the Submitting Author has the right to grant and does grant on behalf of all authors of the Work (as defined in the below author licence), an exclusive licence and/or a non-exclusive licence for contributions from authors who are: i) UK Crown employees; ii) where BMJ has agreed a CC-BY licence shall apply, and/or iii) in accordance with the terms applicable for US Federal Government officers or employees acting as part of their official duties; on a worldwide, perpetual, irrevocable, royalty-free basis to BMJ Publishing Group Ltd ("BMJ") its licensees and where the relevant Journal is co-owned by BMJ to the co-owners of the Journal, to publish the Work in this journal and any other BMJ products and to exploit all rights, as set out in our licence.

The Submitting Author accepts and understands that any supply made under these terms is made by BMJ to the Submitting Author unless you are acting as an employee on behalf of your employer or a postgraduate student of an affiliated institution which is paying any applicable article publishing charge ("APC") for Open Access articles. Where the Submitting Author wishes to make the Work available on an Open Access basis (and intends to pay the relevant APC), the terms of reuse of such Open Access shall be governed by a Creative Commons licence – details of these licences and which Creative Commons licence will apply to this Work are set out in our licence referred to above.

Other than as permitted in any relevant BMJ Author's Self Archiving Policies, I confirm this Work has not been accepted for publication elsewhere, is not being considered for publication elsewhere and does not duplicate material already published. I confirm all authors consent to publication of this Work and authorise the granting of this licence.

For peer review only

The measurement and outcomes of co-production in health and social care - a systematic review of empirical studies

Abstract

Background: Co-production is promoted as an effective way of improving the quality of health and social care but the diversity of measures used in individual studies makes their outcomes difficult to interpret.
Objective: The objective is to explore how empirical studies in health and social care have described the outcomes of co-production projects and how those outcomes were measured.
Design and methods: A scoping review forms the basis for this systematic review. Search terms for the concepts (co-produc* OR coproduc* OR co-design* OR codesign*) and contexts (health OR 'public service* OR "public sector") were used in: CINAHL with Full Text (EBSCOHost), Cochrane Central Register of Controlled trials (Wiley), MEDLINE (EBSCOHost), PsycINFO (ProQuest), PubMed (legacy) and Scopus (Elsevier). There was no date limit. Papers describing the process, original data and outcomes of co-production were included. Protocols, reviews and theoretical, conceptual and psychometric papers were excluded. The PRISMA guideline was followed. The Mixed Methods Appraisal Tool underpinned the quality of included papers.
Results: 43 empirical studies were included. They were conducted in 12 countries, with the UK representing more than 50 % of all papers. No paper was excluded due to the Mixed Method Quality Appraisal screening and 60 % of included papers were mixed method studies. The extensive use of self-developed study-specific measures hampered comparisons and cumulative knowledge-building. Overall, the studies reported positive outcomes. Co-production was reported to be positively experienced and provided important learning.
Conclusions: The lack of common approaches to measuring co-production is more problematic than the plurality of measurements itself. Co-production should be measured from three perspectives: outputs of co-production processes, the experiences of participating in co-production processes, and outcomes of co-production. Both self-developed study-specific measures and established measures should be used. The maturity of this research field would benefit from the development and use of reporting guidelines.

Article summary

Strengths and limitations of the study

- This systematic review embraces co-production within both health and social care, which facilitates a holistic understanding of co-production from a user perspective.
- This systematic review regards empirical studies, indicating that identified measures are relevant and possible to use practically, in empirical settings.
- Only peer reviewed research published in English is included, which may mean that important co-productions research published in other languages has been overlooked.

For peer review only

Key words: Co-production; Evaluation; Measurement, Methods, Outcomes; Systematic review

The measurement and outcomes of co-production in health and social care - a systematic review of empirical studies

Introduction

Despite the term co-production stemming back to Ostrom's seminal work in the 1970s [1], the study of co-production is nonetheless sometimes viewed today as having low scientific maturity [2-4]. For the purposes of this review – and following others [5]- we include co-design with its origins in the participatory design movement in Scandinavia also in the 1970s as a specific form of co-production. Co-production research has been reported as typically not outcome-focused, tending rather to describe co-production processes [3, 6]. The concepts of co-production and co-design are attracting increasing research interest [2, 7], albeit whilst appearing to labor under a multitude of definitions [7]. This systematic review adopts the definition proposed by Osborne et al.; 'the voluntary or involuntary involvement of public service users in any of the design, management, delivery and/or evaluation of public services' [5, p 640].

This systematic review is part of the Samskapa research program on co-production [8] and concerns the measurement and outcomes of co-production as it relates to the provision of both health and social care services. Health and social care are provided by various organisational types in different countries, including private, public and third sector organisations. Such services aim to improve social or health-related aspects of citizens' lives and are important for quality of life [9]. An example, the application of co-production in health and social care services is the creation, further development and evaluation of a smartphone app for young people with lived experience of self-harm [10]. To maximize the use and acceptance of the app, product users (young people with lived experiences of self-harm and clinicians), researchers and app developers collaborated through all stages of this project. The increasing number of such empirical studies of co-production in these sectors is predominantly in health care; of the ten most cited papers on co-production, none concern social care [2]. Given widely held ambitions to integrate health and social care services [9], this focus is problematic, and thus, this systematic review embraces both health and social care services.

Advocated as an effective way of improving quality of health and social care [11-13], co-production is also considered more broadly as a means to promote a democratic ethos placing equal value on the contributions of both citizens and service providers in the successfully delivery of services [12, 14]. The benefits of Patient and Public Involvement (PPI) in research is also acknowledged as a moral obligation with several potential benefits [15].

There are several plausible explanations related to the challenges researchers encounter when seeking to measure the outcomes of co-production [16], including its emergent and adaptive nature which can impede the early determination of appropriate measurements and which might otherwise neglect outcomes that are significant to

For peer review only

participants [17]. Co-production also often encompasses complex and broad needs [18], and is characterized by both a multitude of stakeholders and a plurality of contexts; such features tend to lead to weak or indistinct cause-effect relationships between co-production activities and their outcomes [19]. However, knowledge of co-production outcomes is important in order to enable the continued improvement of services [20]. In summary, whilst important to explore the outcomes of co-production, these are often difficult to measure through a priori measures and deductive analysis [6, 21, 22], and to date methodological diversity has been limited [13].

Regardless of these challenges, scholars have previously used various categorisations to present outcomes, further complicating attempts to synthesize them and build a broader evidence base. Examples of categories of co-production outcomes include: outputs and outcomes [11], discrete products, care processes and structural outcomes [23], patient/staff involvement, generating ideas and tangible change [24] and both individual and collective outcomes [4]. Alternatively, innovation potential, individual well-being and citizen empowerment, increased effectiveness and efficiency, mobilization of resources and increased democracy are categories suggested by Brix et al. [19]. Other types of similar broader outcomes include increasing effectiveness, increasing citizen involvement, greater efficiency, gaining customer satisfaction, strengthening social cohesion and democratizing public services [3].

There are a small number of reviews appraising the research designs and methods of co-production across the public sector. In their systematic review on empirical studies on co-production and co-design e.g., Voorberg and colleagues report that qualitative case studies, single or comparative, predominate [3]. More robust critiques of the designs and methods typically employed to study the outcomes of co-production may help to improve the scientific maturity of this research field and to enable better understanding of the wide range of reported outcomes. In this systematic review, outcomes are understood as qualitative and quantitative descriptions used to capture the impact, effect, results, or outputs of ongoing, or implemented, co-production projects. Objectives, measures and outcomes are interrelated. Outcomes are described by the use of measures, and evaluated in relationship to stated objectives. In seeking to explore the measures and outcomes of co-production, knowledge of the project objectives is necessary.

The aim of this systematic review is to explore how empirical studies in health and social care have described the outcomes of co-production projects and how such outcomes have been measured.
The review questions are:

- What are the objectives of co-production projects?
- What measures have been used?
- What are the outcomes of co-production?

Methods and materials

In the published Samskapa study protocol the aims were to explore, enhance and measure the value of co-production for improving the health and social care of citizens [8]. A scoping review was performed as part of this wider study in March 2019 [7], and the included articles form the basis for this systematic review. This systematic review

For peer review only

has followed the Preferred Reporting Items for Systematic Reviews and Meta-Analyses, the PRISMA guidelines [25], and the manuscript is organised according to the instructions for authors.

Information sources, eligibility criteria, search strategy and selection process for the scoping review

The scoping review set out to explore 'what is out there' in the peer reviewed research relevant to co-production or co-design in health and social care services [7]. The full search strategy is provided in supplemental File 1. In brief, search terms for the concepts (co-produc* OR coproduc* OR co-design* OR codesign*) and contexts (health OR 'public service* OR "public sector") were used in the following databases: CINAHL with Full Text (EBSCOHost), Cochrane Central Register of Controlled trials (Wiley), MEDLINE (EBSCOHost), PsycINFO (ProQuest), PubMed (legacy) and Scopus (Elsevier). There was no specified date limit. Articles were included in the scoping review if they related to peer-reviewed research, consisted of any methodology relevant to co-production or co-design in the context of health and social care services, involving service-users and were written in English. The results were stored in a database in Excel and in Rayyan, a web- and mobile app for systematic reviews that helps expedite the initial screening of abstracts and titles using a semi-automation [26].

Eligibility criteria and selection process for this systematic review

The 979 records from the scoping review were screened initially by one of the researchers (D.M), to remove papers without empirical data or explicit references to measurements. The titles and abstracts of identified papers were added into a new Rayyan data base and reviewed by two of the researchers (AN and KAJ), who independently noted 'include', "unsure" or 'exclude' in Rayyan based on the three additional eligibility criteria for this systematic review:

1. Process of co-production is described (inclusion criterion)
2. Original data from process and outcomes is described (inclusion criterion).
3. Not a theoretical, conceptual, protocol, review or psychometric paper (exclusion criterion)

Conflicting decisions, or decisions the researchers were undecisive of, were crosschecked and validated by the two researchers and remaining conflicts were discussed until consensus was reached. The eligibility criteria were then applied on the included full text papers. To enhance the reliability of the review process, the two researchers read the same first five papers and discussed their decisions until consensus was reached. Another five papers were reviewed following the same procedure and thereafter, remaining papers were examined separately, with decisions noted in a shared Excel file. The quality of each included papers was then appraised.

The Mixed Method quality Appraisal Tool (MMAT) has been developed to assess the methodological quality of empirical studies [27] and was used to appraise the quality of included full text papers. MMAT includes five study designs (qualitative, randomized controlled trial, non-randomized, quantitative descriptive and mixed methods) and for each study design, five criteria for the evaluation of the methodological quality are provided. [28]. The criteria can be evaluated 'yes', 'no' or 'can't tell', and affirmative responses indicate quality. Two initial screening questions are included to assess

For peer review only

whether the study is empirical. The first five papers were assessed jointly by two researchers (AN and KAJ). Another five papers were assessed separately and the results compared and discussed. On the basis of the MMAT tool, the remaining papers were assessed independently and the results documented in a shared Excel file. Two meetings of the researchers were held to discuss queries. The MMAT study designs are used as categories to group the included studies in the synthesis of our findings below.

Data collection process and data items for this systematic review

To develop a shared work procedure for the data collection two researchers (AN and KAJ) jointly extracted data from five papers and compared and discussed the results. The remaining data extraction was carried out independently by the two researchers and documented in separate files, one for each MMAT category. To enhance consistency, data extraction definitions were inserted as column headings in the Excel files. A count of the number of papers within each MMAT study design category was made. A count was also completed for each of the study design characteristic variables and the proportion of affirmative responses on the MMAT screening questions. After completion of the data extraction, the findings were again discussed with PhD students in the Samskapa research program. The discussion was intended to provide a fuller understanding of the meaning of the results for our future work in the wider program. A plain language blogpost including discussion points inviting comments from a wider audience was also written [29].

In Table 1, the research questions are presented alongside the definitions for data extraction.

Table 1. Research questions and data extraction definitions.

**Research questions**	**Data extraction definitions**
What are the objectives for co-production projects?	Descriptions of the initiators of the projects and the aims of the studies.
What measures are used?	Descriptions of used measures (scales, questionnaires or other forms of measurements).
What outcomes of co-production are reported?	Qualitative or quantitative descriptions of the impact, effects, results or outputs of ongoing or implemented co-production projects, including learning and social and cultural effects.

In Table 2, other variables for which data was sought are listed and defined.

Table 2. Variables for study characteristics and data extraction definitions.

**Study characteristics**	**Data extraction definitions**
Year	Year of publication
Country	Country in which the study was conducted
Field	The specific health- or social care field for the study

For peer review only - http://bmjopen.bmj.com/site/about/guidelines.xhtml

Synthesis methods

The extracted data in each MMAT study design category was analysed separately. In the first step of analysis, the data extractions were closely read. In the next step, the data extractions formed the basis for a convergent qualitative synthesis [28]. A synthesis is a process of putting the findings from individual studies together into a new or different arrangement and thus providing knowledge that would not otherwise be apparent while reading the individual studies in isolation [30]. The measures were analysed and coded into four categories 1) International validated scales (including scales with minor adaption or use of subscales), 2) Measures based on publications (e.g. based on public documents, published research, indicators and non-validated scales), 3) Self-developed study-specific measures (locally developed measures), and 4) Measures on the experience of co-production.

Patient and public involvement

No patient involved.

Results of the systematic review

Study selection

The first initial screening of all papers included in the scoping review reduced the number of potential papers from 979 to 187. Thereafter the titles and abstracts of the remaining papers were reviewed and 116 papers were excluded. Thirty-seven papers were excluded based on the first eligibility criteria, 34 papers based on the second eligibility criteria and 45 based on the third eligibility criteria. The eligibility criteria were then applied on the included full text papers, leading to the exclusion of a further 28 papers. Fifteen papers were excluded based on the first eligibility criteria, 8 papers based on the second eligibility criteria and 5 based on the third eligibility criteria. The remaining 43 papers formed the basis for the quality appraisal and the data extraction for this systematic review. The PRISMA flow diagram is based on the PRISMA 2020 flow diagram template for systematic reviews [31], and is presented in Figure 1.

Figure 1. The PRISMA flow diagram

Please insert figure 1 here

Result of the quality assessment

Of all included papers in the systematic review, 86% were considered as having a clear research question (screening question No 1 of MMAT) and 95% to have a relevant data collection process (screening questions No 2 of MMAT). The largest proportion of included papers (60%) reported on studies with mixed method study designs (Table 3). The distribution of affirmative responses to the MMAT categories indicate the general high quality of the included studies.

Table 3. Proportion of affirmative categories in MMAT per study design.

	Yes on 5 MMAT criteria	Yes on 4 MMAT criteria	Yes on 3 MMAT criteria	Yes on 2 MMAT criteria	Yes on 1 MMAT criteria	Yes on 0 MMAT criteria

For peer review only

**Qualitative studies** 12 % (5)	60 % (3)	0 % (0)	40 % (2)	0 % (0)	0 % (0)	0 % (0)
**Quantitative randomized controlled trials** 7 % (4)	25 % (1)	0 % (0)	25 % (1)	25 % (1)	25 % (1)	0 % (0)
**Quantitative non-randomised studies** 7 % (3)	0 % (0)	33.3% (1)	33.3 % (1)	0 % (0)	0 % (0)	33.3 % (1)
**Quantitative descriptive studies** 14 % (6)	50 % (3)	33 % (2)	0 % (0)	17 % (1)	0 % (0)	0 % (0)
**Mixed methods studies** 60 % (25)	40 % (10)	24 % (6)	16 % (4)	12% (3)	8 % (2)	0 % (0)

Study characteristics

The studies located in this review were published between 2012 and 2019 (online Supplemental Figure 1). The year 2018 saw the most papers (n=17), however only the first three months of 2019 are included.

The 43 studies were conducted in 12 different countries, generally with three or fewer studies per country. The exceptions are the United Kingdom (n=23) and Australia (n=6). The studies were conducted in different fields of health and social care; mental health has the largest proportion of studies (n=14). The UK represents 53% of the included studies and of the 23 studies conducted there, 9 related to a mental health setting.

Based on our convergent design [28] our findings are reported below for each research question in turn. In the text, user is used as a generic term for those who are using a service, innovation or healthcare innovation in health and social care. The term informal caregiver is used as a generic term for individuals supporting and helping the users, based on personal engagement (and not a professional responsibility) and providers is used as a generic term for those who are providing a service. In Appendix 1 (online Supplemental File 2) the outcomes and measures are compiled.

Results of syntheses

What are the objectives of co-production projects?

The objectives were explored by analysis of who initiated the co-production projects and establishing the aims of the study. Users rarely initiated co-production projects or studies.

The aims of the *qualitative studies* ranged from increasing communication with users to encouraging service innovation in partnership with them. Some of the studies also aimed to increase users' engagement with health and social care services and to assess how users and providers perceive services and experience quality [32]. In the few cases the origins of the studies were described, the studies had been initiated by researchers.

All the *quantitative randomized controlled trials* aimed to evaluate outcomes of co-produced interventions. Two studies aimed to provide better, more timely support through the use of digital tools [10, 33]. One study aimed to increase the patient and public involvement in research [34] and a further study aimed at improving staff behaviours and actions to improve care through co-produced interventions [35]. Those who initiated the projects - and their reasons for doing so - were not described in the studies.

Descriptions of the initiators of project were only included in two of the three *quantitative non-randomized controlled studies*. The project by Hahn-Goldberg et al. [36] was initiated by the hospital management after a co-produced pilot study and the project by Bolton et al. [37] was initiated by several organisations aiming to promote public health. All three studies aimed at improving users´ experiences of provided services [36-38].

None of the included *quantitative descriptive studies* described users as having initiated the projects. Chapman et al. [39] aimed at evaluating a co-produced plan to reduce emergency visits. Lamph et al. [40] aimed at evaluating co-production in an e-learning program to improve staff attitudes. Turner et al. [41] aimed at evaluating a co-designed and co-produced program to improve patients' health and self-management skills and Roberts et al. [42] aimed at improving care through individualized care quality data. Some studies aimed at increasing co-production within processes. Cramm et al. [43] aimed at developing the care process by improving the co-productive relationship. Adinolfi et al. [18] aimed to improve co-production processes and patient outcomes by integrating health and social care and increasing user empowerment.

The aims of the *mixed methods studies* varied. One study aimed to empower disadvantaged communities [44] and one aimed at testing co-production in areas with high levels of violence [45]. Other aims were to evaluate the experience of partaking in co-design [46] and to evaluate how co-design can improve patient experiences and services [47]. Four studies aimed at improving co-production within already ongoing work processes [48-51]. Most of the studies aimed at evaluating outcomes of co-produced information, interventions/services or products [47, 48, 52-67]. Among the studies evaluating outcomes of co-produced interventions/services the focus was on effectiveness [52, 61] and on the level of improvement [54]. Evaluation of acceptability and feasibility of co-produced interventions or products was the aim of several studies [58, 65, 67] and two studies aimed at evaluating the use and experience of a co-designed product [59, 60]. Other studies aimed at evaluating user-led co-produced interventions [63, 66]. However, of the 25 mixed method studies only one stated that the study was initiated by users [56]. Also, only one study compared a co-produced method with other methods [68].

What measures are used?

In the *qualitative studies*, no measures beyond simple descriptive calculations were used.

In the *quantitative randomized controlled trials* several validated scales were used. Two studies used three different validated scales each [10, 35] and one [33] used two validated scales. The scales focused on health- or clinical aspects related to the

For peer review only

interventions. Two self-developed study-specific measurements purposing to evaluate the intervention itself were used [10, 34, 35] and in the study by Hasting et al. [35] a scale based on previous publications was used.

Self-developed study-specific measurements focusing on the experiences of an intervention and the implementation process were used in the *quantitative non-randomized controlled* studies [36-38]. In the study by Bolton et al. [37] two validated scales focusing on health aspects related to the intervention and three questionnaires based on other publications were also used. It is worth noting that the validity and reliability of the outcome measurements were rarely discussed. The focus was more on the sustainability and usefulness of the innovations/interventions.

In three of the six *quantitative descriptive studies* validated scales were used. Turner et al. [41] used five different validated scales. Cramm et al. [43] one study used two validated scales and Adinolfi et al. [18] used one validated scale. The scales focused on health or clinical aspects related to the interventions. Two studies included self-developed study-specific measurements concerning the interventions [18, 39] and one study included self-developed measurements regarding the clinical outcomes [42]. Three studies used measurements based on previous publications [18, 40, 42]. Cramm et al. [43] used The Relational Coordination Scale (RCS) to assess participants' perceptions of interaction productivity, thus measuring the experience of the co-production process itself.

Seven of the 25 *mixed method studies* used validated scales. In three studies, one validated scale was used [51, 55, 67]. Three studies used two different validated scales [48, 58, 65] and Ferguson et al. [49] used three different validated scales. Two of the studies used scales that, unlike the others, did not focus on health or clinical aspects. Instead, they used validated scales measuring the quality [55] and usability of technical systems or innovations [51]. Eleven studies used one or more measures based on previous research [45, 47, 52-58, 61, 67]. Five studies did not use any self-developed study-specific measures [47, 48, 56, 57, 67]; all others did. Two studies used measures regarding the experience or level of the co-production process. Revenäs et al. [46] used a self-developed survey to assess users' experience of participating in the co-production process, and Haynes et al. [56] measured how the intervention adhered to the National Health and Medical Research Council's (NHMRC) principles. Brosseau et al. [50] did not measure the level of co-production but referred to several ways this could have been done. Morales-Peres et al. [45] also did not measure the co-production level; however, they concluded that the study results were made possible due to the high levels of participatory engagement.

What outcomes of co-production are reported?

A wide variety of outcomes were presented in the *qualitative studies,* commonly describing the number of innovations or implemented changes, the establishment of priority areas or consensual agreement and the development of a tool [32, 69-72]. The sustainability of outcomes was presented as having to encompass financial sustainability, new organisational relationships and further quantitative studies [70]. Organisational structures and issues related to patient safety were described as potentially hampering the implementation of digital tools, thus influencing sustainability negatively [32].

The outcomes in the *quantitative randomized controlled trials* were positive for two interventions targeting users, showing significant reduced self-harm and reduced mental ill-health, and that the users wanted to continue using the co-produced digital tools [10, 33]. A third study targeting mental health patients did not report any positive outcomes [34]. The outcomes for interventions targeting providers were also favourable, but the results were not as strong as in the studies targeting users [35].

Regarding the *quantitative non-randomized controlled studies,* Hahn-Goldberg et al. [36] presented outcomes related to the use of a new tool, but the effects of the tool, including the costs are yet to be evaluated. With the objective of identifying the vital outcomes of the project, Murphy et al. [38] used several measurements covering different perspectives. The measurements concerned outcomes of the process itself, user surveys and user narratives. Some of the surveys aimed at family members had response rates too low to provide useful data. However, narratives from users were used to complement the survey data and these data showed both positive and sustainable outcomes. The research teams concluded that the value of collaborating with stakeholders to find useful measurement and tools for assessments was an important learning outcome. Bolton et al. [37] used measures not tested beyond their face validity but there were other outcomes showing positive results such as new social support networks and user requests for support in gaining knowledge supporting their health.

Cramm et al. [43] and Lamph et al. [40] described quantitative results and correlations from established questionnaires in their *quantitative descriptive studies.* However, Lamph et al. [40] also included observational process outcomes. Turner et al. [41] additionally included health data, registry data and observational process outcomes. Chapman et al. [39] described quantitative results of objective measurements and Roberts et al. [42] described compliance with clinical practices and quality indicators. Quantitative outcomes in terms of individual health status and cost savings on organisational levels were also reported by Adinolfi et al. [18].

The outcomes in the *mixed method studies* were extensive and to facilitate an overview the outcomes are presented below in four broad themes: effects on the outcomes and processes, success factors, impact on participants and learning outcomes.

Outcomes and processes

Co-production led to many effects on the outcomes and processes the projects aimed to improve, with improved care [62], stronger social networks [63] and increased urgency in communication as [56] some examples. Co-production with users indicated what worked well and what needed to be improved [48, 66] and combining quality improvement and co-design led to additional quality improvement projects [50]. Co-produced products like Reusable Learning Objectives were highly cost-effective and showed significant effects on outcomes, knowledge, skills, uptake and adherence. Reusable learning Objectives were also highly rated as useful and recommended to others [49]. Studies measuring outcomes during the development of a technical product reported that co-production with users was helpful, and that it provided understanding valuable for the further improvement of the product [55, 59, 67]. In one study the product uncovered unmet health needs among participants [55] and in another study,

For peer review only

the final testing revealed that a co-produced product had led to improved symptoms [60]. One study resulted in a non-functioning IT product [51].

There were positive effects of using co-production to improve processes [54, 55, 57, 58, 65], and the combination of quantitative and qualitative measurements led to a greater understanding of the components influencing the results [54, 58, 65] and additional increased engagement by families [54]. The results also specified requirements for further development of tested interventions [58]. D'Young et al. [61] reported that co-production lead to the fulfilment of the effectiveness goals of a service.

Success factors

Close engagement and co-production with those intended to take part in, or benefitting from the intervention, were identified in several *mixed methods studies* as success factors [44, 45, 48]. Another identified success factor was the use of emancipatory process indicators (for example the use of an emoticon survey on important values) in the implementation and evaluation of projects [56] and facilitator training [45].

Impact on participants

Co-production was an influential experience for participants and organisations [45, 48, 52, 53, 66]. Improved well-being [63], respect and confidence [45], and empowerment [56] were examples of influential experiences. However, participation in co-production projects also lead to paradoxical experiences as a wish to engage more participants, while preferring to work in separate groups: a desire for more preparation and discussion, while considering invested time as a concern; and the view on co-design as valuable for improved care, while doubting the realization of co-care in practice [46]. The participation also led to feeling of uncertainty and vulnerability in one study [63]. In their study Revenäs et al. [46] suggested that co-design methods influenced the participants' experiences of the process, thus concluding that such methods need to be adjusted to participating stakeholders and contexts.

Learning outcomes

Co-production led to several unforeseen learning outcomes and learned principles were highly valuable in the next step of improvement and development [56]. One study resulted in learning outcomes such as the importance of motivation; identification of most functional techniques; evaluation of mock-ups with end-users; and the recognition of the influence of informal caregivers [51]. Similar learning outcomes were also presented as results in other studies. Rosso and McGrath [44] summarized the learning outcomes of their study as the importance of: formatting a regional partnership action group; selecting appropriate communities according to needs and preparedness; engaging community champions in each location; establishing a continuous consultation framework relating to each location and the regional action group; co-designing culturally appropriate age-relevant activities in collaboration with local stakeholders; and considering sustainability. Boyd et al. [47] summarized the learning outcomes of their study as the value of using co-design alongside traditional quality improvement methodologies; early engagement with patients; staff buy-in; and individuals trying things outside their comfort zones. Another study brought forward methodological learning outcomes, i.e., the synergy between Lean and Experience-Based Co-Design [50]. Only two studies with a mixed methods design measured sustainability over time [52, 64]. Notable, the only study comparing co-production with other methods, stated that

the data gathered (in this case, patient incident reports) was unlikely to have been found through other established methods [68].

Analysis and discussion

General interpretations of results

Knowledge of the outcomes of coproduction is fundamental for assessing the value of co-production projects, and to inform approaches to continually improve services important to citizens. Overall, the studies included in this systematic review showed positive outcomes from co-production but these were difficult to compare across the studies. One plausible explanation for this lies in the ambiguous nature of the various ways in which co-production is conceptualised. Co-production is, and has been, defined and applied in multiple ways in health and social care [7]. Nonetheless, co-production was a positive experience for participants in our included studies and the findings provided learning important to enable a range of stakeholders to continuously engage in such processes. Various measures were used, and the outcomes were presented in a diversity of categorisations. The results of this systematic review point at three perspectives that are important for measuring the outcomes of co-production. These perspectives, and observations regarding study designs are discussed below.

Perspective 1 – Outputs of co-production processes

The first perspective concerns outputs. Outputs are the results of the processes that are undertaken to contribute to the stated objectives of a project [73]. When outputs are measured continuously, they can also be used to monitor ongoing work [74]. The extensive use of a range of self-developed study-specific measurements in the included studies limited comparisons and cumulative knowledge-building regarding co-production. Two exceptions were found where the validated measures concerned the quality or usability of the intervention itself i.e., outputs of the co-production process. The Mobile Application Rating Scale (MARS) was used to explore users' acceptance and experience of a co-produced App [55] and the System Usability Scale (SUS) was used to assess user experience of a mobile App [51].

A precondition for identifying relevant measures in relation to outputs is that the processes are carefully described, and a fundamental aspect of co-production processes concerns how and when users are involved. In general, these processes have been described in detail; however, the clarity concerning in which phases of the process users participated could have been clearer in many of the studies. Without this, studies appear to have been co-produced even when participation was restricted to only one phase. In their generic program theory for co-production, Brix et al. [19] suggest three phases (co-design/co-planning, co-delivery/co-creation and co-review/co-evaluation) which may be helpful to clarify when and how users participate.

Perspective 2 - Experiences of participating in co-production processes

The second perspective concerns participants' experiences of participating in co-production. Given that co-production is anchored in democratic and equal value principles [12, 14] it is notable that only three studies provided insights into how these core values might be measured. The Relational Coordination Scale (RCS) scale was used to assess how participants perceived the interaction among actors during the

collaboration [43] and the National Health and Medical Research Council's (NHMRC) principles guided a strong focus on values important for collaboration with indigenous communities [56]. The self-developed questionnaire by Revenäs et al. [46] measured how participants experienced the possibility of having their voice heard during a co-production collaboration. In general, users' objectives to participate were not well described. As their participation is fundamental for co-production, this is notable. If the initiative takers are unaware of the users' intrinsic and extrinsic aims for participation, they can neither take them into account nor create receptive conditions for them to contribute. There is a need to take users' objectives for participating into explicit account, and to measure experiences of participation.

Perspective 3 - Outcomes of co-production

The third perspective concerns the interventions' stated objectives and expected outcomes [11]. Twenty-six of the 28 validated scales in this systematic review involved this perspective and covered different aspects of improved health or better clinical outcomes. Overall, the studies reported positive outcomes. However, there is a difference between positive outcomes and goal fulfilment. Various kinds of measures were used but the goal levels for them were seldom set before projects started. Users were seldom involved in the choice of measures or decisions on goal levels. This lack of shared operational objectives makes it difficult to evaluate if the co-production projects reached their expected outcomes – especially from the perspective of users - and has to our knowledge been less problematized in the literature than the lack of shared measurements per se.

Domination of mixed method studies

Mixed method study designs are well-suited for studying complex phenomena such as changes in behaviours or experiences from different perspectives [75]; 60% of the studies in this systematic review used different mixed method study designs. The use of mixed method study design in research in health and social care is increasing [76] but qualitative study designs are typically the most common study designs in co-production research [77]. In qualitative studies, measurements do not have the same central role as in quantitative studies and in the mixed method studies included in this systematic review, qualitative methods were prominent. The study designs identified in this systematic review are in line with both the general trend towards more mixed study designs in health and social care, and the use of qualitative approaches in co-production.

Implications for practice

In this systematic review, we did not identify any generic measures of the outcomes of co-production and nor would we recommend such an approach. Instead, we suggest a model facilitating comparisons between studies and projects that does not limit flexibility and can incorporate the use of both self-developed study-specific measures and established measures. In complex contexts, cause-and-effect relationships are not linear and unexpected consequences are common. To address this, a process for selecting a small number of measures representing different perspectives has been suggested [78, 79]. Balanced score card [80] and the value compass [78] are examples of models that seek to help pinpoint different perspectives which are important for measuring and monitoring organisational performance and processes.

For peer review only

We propose a model for balanced measurement of co-production (Figure 2) which combines output measures (Perspective 1) and outcome measures (Perspective 3). Our emphasis on continuous output measures is in line with a continuous quality improvement approach since this clarifies *how* the processes or interventions works. Both perspective 1 and 3 are important to monitor and evaluate change [79]. The model also aligns with the core values of co-production by incorporating how participants experience their own participation in the co-production process (Perspective 2). The model proposes the use of at least one measure per perspective, and the use of both established and self-developed study-specific measures. The chosen measures should be selected to offer actionable information for further improvements.

Figure 2. A model for the balanced measurement of co-production.
Please insert figure 2 here

The notion of "balanced" in this context refers to the act of weighing the outcomes together. A balanced approach may prevent co-production from being used to achieve project objectives without addressing its democratic principles. It may also help focus attention on co-production projects in which participants report positive experiences but without the project objectives being fulfilled. Based on how we have found co-production to have been reported in the 43 studies in this systematic review, the model is a radical proposition; only Adinolfi et al. [18] have measures reflecting all three proposed perspectives.

The model also emphasizes the use of both established measures (Appendix 1, first two columns, online Supplemental File 2) and self-developed study-specific measures (Appendix 1, third column, online Supplemental File 2). Established measures (internationally validated scales and measures based on publications) are depicted as a light-grey core at the centre of the model. This core of measurements corresponds with the outcome measures suggested by Marsilio et al. [77] for example: validated scales, other metrics, and indicators. In our systematic review a multitude of measures based on publications were used whereas the use of internationally validated scales was much more restricted. This may indicate a lack of knowledge of existing validated scales. Broadened multidisciplinary competence among researchers and professionals in the field of co-production may support a greater use of validated scales, which could then strengthen the reliability of results and promote comparisons. The model also includes self-developed study-specific measures, placed in the dotted zone surrounding the core (Figure 2). Such measures are "tailor-made" for the specific purposes of a single study and their joint development by users and providers within a study can be a foundation for shared understanding, creativity and engagement.

Our proposal for self-developed study-specific measures is in line with the findings in Greenhalgh et al's systematic review regarding frameworks for patient and public involvement [81], in which they state that it might be more efficient and sustainable to develop one's own framework than to apply someone else's. Another implication from Greenhalgh's et al review relating to our suggested model is the need for the actors themselves to formulate operational objectives, so that they - with their own measures - can determine whether the co-production project has reached its intended goals. Finally, included papers in this systematic review generally showed a low awareness regarding the difference between outputs and outcomes, and we believe that the proposed model

For peer review only

can clarify both the differences between the two perspectives, and their shared importance.

Implications for future research

An important implication for research concerns the need for closely described co-production processes, including clarifications of the activities, or process steps, that users participate in. Such clarity would provide better understanding of how co-production activities impact outcomes. A good example of how to make the co-production process in research transparent is provided by Marks et al. [82]. In their scoping review, they list specific citizen science activities and group them into categories describing increasing public involvement (contributory, collaborative, co-created and citizen- lead).

A second implication for future research concerns the composition of project groups, which in general need to include more multidisciplinary competences. A broader knowledge base can facilitate the identification and use of established measures, thus linking local co-production projects to a cumulative body of knowledge. A third implication concerns the description of measures. In several papers authors refer to measures, surveys or survey questions without describing them. Regardless of the type of measures, these should be described in sufficient detail that researchers can consider them for other studies. A fourth implication concerns the need for better knowledge of which mixed method study designs are most suited for research in co-production in health and welfare services. Lastly, the diversity in how co-production research was reported in the papers included in this systematic review points to a major implication, the development of reporting guidelines for co-production research. The adoption and use of such a guideline would significantly contribute to the scientific maturity of the research field.

Strengths and limitations

This review is in line with the published study protocol [8] and contributes to the aim of exploring, enhancing and measuring the value of co-production for improving the health and social care of citizens. Against the background of the widespread ambitions to integrate health and social care services, whilst the number of empirical studies on co-production is increasing in health care, it is a strength that this systematic review includes both health and social care services. Furthermore, the MMAT tool was crucial for appraising the quality of included studies and provided an overview of the used study designs. The categories used to code and analyse the extracted measures were also easy to use and can be recommended to other studies and projects on co-production. The identified measures are empirically tested which indicates that they are relevant and possible to use in other empirical settings.

The data for this systematic review was obtained from a scoping review, in which only peer review research, published in English were included [7]. Though justified considering the aims of this systematic review, this inherently narrowed our focus and may mean we have missed important applied co-production research published in other languages. We also acknowledge that it would be ideal to update the review. However, the detailed analysis was a time-consuming process and repeating the analysis would create a similar delay between an updated search and publication. Further to this, research exploring Cochrane reviews described that only a minority were updated to

For peer review only

incorporate evidence from new primary studies. Of those that did, very few updates led to changes in the conclusions [83]. We believe this applies to our systematic review as well and that the overall findings are most unlikely to change with an update covering only a small number of years The more recently published reviews with which we have compared our results lend weight to this belief.

Conclusions

The objective of this systematic review was to increase understanding of how empirical studies have measured the outcomes of co-production and co-design in health and social care and what the outcomes are. Overall, the studies reported positive outcomes using a multitude of measures. This plurality limited comparisons and cumulative knowledge building but the lack of common strategies and models for measuring co-production is more problematic than the plurality itself. The findings of this systematic review suggest that co-production should be measured from three perspectives: the outputs of co-production processes, the experiences of participating in co-production processes and outcomes of co-production. Two types of measures should be used: self-developed study-specific measures and established measures. By measuring and monitoring co-production with this balanced approach the democratic aspects of co-production and its complexity can be incorporated within future evaluations. By using the model for the balanced measurement of co-production, we believe that future studies in co-production can better contribute to "the science of participation" [84]. The development of a reporting guideline would also enhance the needed transparency and the scientific maturity of this research field.

Contributor ship statement

Authors confirm contribution to the paper were as follows: all authors (AN, SK, GR, DM and KAJ) took part in the study conception and design. AN, DM and KAJ performed the data collection. AN and KAJ performed the data extraction, analysis and interpretation of results. AN and KAJ made the draft manuscript preparation. DM and AN wrote the method section and AN wrote the discussion section. All authors (AN, SK, GR, DM and KAJ) reviewed the results and approved the final version of the manuscript. We are grateful for the help from all participants in co-production events, from our university library. Also, thanks to colleague B.L, who engaged in the improvement of Figure 2.

Competing interests

The authors declare no conflict of interest.

Funding

This work is part of the Samskapa program which is supported by Forte: Forskningsrådet om Hälsa, Arbetsliv och Välfärd, grant number 2018-01431.

Data sharing statement

Data are available upon reasonable request.

Ethics approval

Not applicable

Abbreviations

MMAT: Mixed Method quality Appraisal Tool

For peer review only

PRISMA: Preferred Reporting Items for Systematic Reviews and Meta-Analyses

For peer review only

References

1. Robert G, Donetto S, Williams O. Co-designing healthcare services with patients. The Palgrave Handbook of Co-Production of Public Services and Outcomes: Springer; 2021. p. 313-33.
2. Fusco F, Marsilio M, Guglielmetti C. Co-production in health policy and management: a comprehensive bibliometric review. BMC Health Services Research. 2020;20:1-16. doi: 10.1186/s12913-020-05241-2.
3. Voorberg WH, Bekkers VJ, Tummers LG. A systematic review of co-creation and co-production: Embarking on the social innovation journey. Public Management Review. 2015;17(9):1333-57. doi: 10.1080/14719037.2014.930505.
4. Loeffler E, Bovaird T. User and community co-production of public services: What does the evidence tell us? International Journal of Public Administration. 2016;39(13):1006-19. doi: 10.1080/01900692.2016.1250559.
5. Osborne SP, Radnor Z, Strokosch K. Co-production and the co-creation of value in public services: a suitable case for treatment? Public Management Review. 2016;18(5):639-53. doi: 10.1080/14719037.2015.1111927.
6. Palumbo R. Contextualizing co-production of health care: a systematic literature review. International Journal of Public Sector Management. 2015;29(1):72-90. doi: 10.1108/IJPSM-07-2015-0125
7. Masterson D, Areskoug Josefsson K, Robert G, Elisabeth N, Kjellström S. Mapping definitions of co-production and co-design in health and social care: A systematic scoping review providing lessons for the future. Health Expectations. 2022. doi: 10.1111/hex.13470.
8. Kjellström S, Areskoug-Josefsson K, Gäre BA, Andersson A-C, Ockander M, Käll J, et al. Exploring, measuring and enhancing the coproduction of health and well-being at the national, regional and local levels through comparative case studies in Sweden and England: the 'Samskapa'research programme protocol. BMJ open. 2019;9(7):e029723. doi: 10.1136/bmjopen-2019-029723.
9. Sullivan H, Williams P. Whose kettle?: Exploring the role of objects in managing and mediating the boundaries of integration in health and social care. Journal of Health, Organisation and Management. 2012;26(6):697-712. doi: 10.1108/14777261211276970.
10. Stallard P, Porter J, Grist R. A smartphone app (BlueIce) for young people who self-harm: open phase 1 pre-post trial. JMIR mHealth and uHealth. 2018;6(1):e32. doi: 10.2196/mhealth.8917
11. Alford J. The multiple facets of co-production: building on the work of Elinor Ostrom. Public Management Review. 2014;16(3):299-316. doi: 10.1080/14719037.2013.806578
12. Holland-Hart DM, Addis SM, Edwards A, Kenkre JE, Wood F. Coproduction and health: Public and clinicians' perceptions of the barriers and facilitators. Health Expectations. 2019;22(1):93-101. doi: 10.1111/hex.12834
13. Verschuere B, Brandsen T, Pestoff V. Co-production: The state of the art in research and the future agenda. Voluntas: International Journal of Voluntary and Nonprofit Organizations. 2012;23(4):1083-101. doi: 10.1007/s11266-012-9307-8
14. Pestoff V. Citizens and co-production of welfare services: Childcare in eight European countries. Public management review. 2006;8(4):503-19. doi: 10.1080/14719030601022882.
15. Baines R, Bradwell H, Edwards K, Stevens S, Prime S, Tredinnick-Rowe J, et al. Meaningful patient and public involvement in digital health innovation, implementation and evaluation: A systematic review. Health Expectations. 2022. doi: 10.1111/hex.13506.
16. Durose C, Needham C, Mangan C, Rees J. Generating'good enough'evidence for co-production. Evidence & Policy: A Journal of Research, Debate and Practice. 2017;13(1):135-51. doi: 10.1332/174426415X14440619792955.
17. Lim S, Morris H, Pizzirani B, Kajewski D, Lee WK, Skouteris H. Evaluating hospital tools and services that were co-produced with patients: A rapid review. International Journal for Quality in Health Care. 2020;32:231-9. doi: 10.1093/intqhc/mzaa020.

For peer review only

18. Adinolfi P, Starace F, Palumbo R. Health outcomes and patient empowerment: The case of health budgets in Italy. Journal of Health Management. 2016;18(1):117-33. doi: 10.1177/0972063415625524
19. Brix J, Krogstrup HK, Mortensen NM. Evaluating the outcomes of co-production in local government. Local Government Studies. 2020;46(2):169-85. doi: 10.1080/03003930.2019.1702530.
20. Lindsay C, Pearson S, Batty E, Cullen AM, Eadson W. Co-production as a route to employability: Lessons from services with lone parents. Public Administration. 2018;96(2):318-32. doi: 10.1111/padm.12408.
21. Greenhalgh T, Jackson C, Shaw S, Janamian T. Achieving research impact through co-creation in community-based health services: literature review and case study. The Milbank Quarterly. 2016;94(2):392-429. doi: 10.1111/1468-0009.12197
22. Filipe A, Renedo A, Marston C. The co-production of what? Knowledge, values, and social relations in health care. PLoS biology. 2017;15(5):e2001403. doi: 10.1371/journal.pbio.2001403.
23. Bombard Y, Baker GR, Orlando E, Fancott C, Bhatia P, Casalino S, et al. Engaging patients to improve quality of care: a systematic review. Implementation Science. 2018;13(1):1-22. doi: 10.1186/s13012-018-0784-z.
24. Clarke D, Jones F, Harris R, Robert G. What outcomes are associated with developing and implementing co-produced interventions in acute healthcare settings? A rapid evidence synthesis. BMJ open. 2017;7(7):e014650. doi: 10.1136/bmjopen-2016-014650
25. Moher D, Liberati A, Tetzlaff J, Altman DG, Group* P. Preferred reporting items for systematic reviews and meta-analyses: the PRISMA statement. Annals of internal medicine. 2009;151(4):264-9. doi: 10.1371/journal.pmed.1000097.
26. Ouzzani M, Hammady H, Fedorowicz Z, Elmagarmid A. Rayyan—a web and mobile app for systematic reviews. Systematic reviews. 2016;5(1):1-10. doi: 10.1186/s13643-016-0384-4
27. Hong QN, Pluye P, Fàbregues S, Bartlett G, Boardman F, Cargo M, et al. Mixed methods appraisal tool (MMAT), version 2018. Registration of copyright. 2018;1148552. doi: 10.3233/EFI-180221.
28. Pluye P, Hong QN. Combining the power of stories and the power of numbers: mixed methods research and mixed studies reviews. Annual review of public health. 2014;35:29-45. doi: 10.1146/annurev-publhealth-032013-182440
29. Areskoug Josefsson K, Nordin A. Insights from co-reflection. Vertikals2020.
30. Denyer D, Tranfield T. Producing a systematic review. In: Buchanan DAaB, A. (Eds), editor. The Sage Handbook of Organizational Research Methods. London: Sage; 2009.
31. Page MJ, McKenzie JE, Bossuyt PM, Boutron I, Hoffmann TC, Mulrow CD, et al. The PRISMA 2020 statement: an updated guideline for reporting systematic reviews. Systematic reviews. 2021;10(1):1-11. doi: 10.1038/nature25753.
32. Sadler E, Porat T, Marshall I, Hoang U, Curcin V, Wolfe CD, et al. Shaping innovations in long-term care for stroke survivors with multimorbidity through stakeholder engagement. PloS one. 2017;12(5). doi: 10.1371/journal.pone.0177102.
33. Bucci S, Lewis S, Ainsworth J, Haddock G, Machin M, Berry K, et al. Digital interventions in severe mental health problems: lessons from the Actissist development and trial. World Psychiatry. 2018;17(2):230. doi: 10.1002/wps.20535
34. Hughes-Morley A, Hann M, Fraser C, Meade O, Lovell K, Young B, et al. The impact of advertising patient and public involvement on trial recruitment: embedded cluster randomised recruitment trial. Trials. 2016;17(1):1-13. doi: 10.1186/s13063-016-1718-1.
35. Hastings R, Gillespie D, Flynn S, McNamara R, Taylor Z, Knight R, et al. Who's challenging who training for staff empathy towards adults with challenging behaviour: cluster randomised controlled trial. Journal of intellectual disability research. 2018;62(9):798-813. doi: doi.org/10.1111/jir.12536.
36. Hahn-Goldberg S, Okrainec K, Damba C, Huynh T, Lau D, Maxwell J, et al. Implementing patient-oriented discharge summaries (PODS): a multisite pilot across early adopter hospitals. Healthcare quaterly. 2016;19(1):42-8. doi: 10.12927/hcq.2016.24610

For peer review only

37. Bolton M, Moore I, Ferreira A, Day C, Bolton D. Community organizing and community health: piloting an innovative approach to community engagement applied to an early intervention project in south London. Journal of Public Health. 2016;38(1):115-21. doi: 10.1093/pubmed/fdv017
38. Murphy L, Wells JS, Lachman P, Bergin M. A quality improvement initiative in community mental health in the Republic of Ireland. Health Science Journal. 2015;9(1):1. doi: hdl.handle.net/10147/560881
39. Chapman H, Farndon L, Matthews R, Stephenson J. Okay to Stay? A new plan to help people with long-term conditions remain in their own homes. Primary Health Care Research & Development. 2019;20. doi: 10.1017/S1463423618000786
40. Lamph G, Sampson M, Smith D, Williamson G, Guyers M. Can an interactive e-learning training package improve the understanding of personality disorder within mental health professionals? The Journal of Mental Health Training, Education and Practice. 2018. doi: 10.1108/JMHTEP-03-2017-0023.
41. Turner A, Realpe AX, Wallace LM, Kosmala-Anderson J. A co-produced self-management programme improves psychosocial outcomes for people living with depression. Mental Health Review Journal. 2015;20(4):242-55. doi: 10.1108/MHRJ-05-2014-0017.
42. Roberts CM, Gungor G, Parker M, Craig J, Mountford J. Impact of a patient-specific co-designed COPD care scorecard on COPD care quality: a quasi-experimental study. NPJ primary care respiratory medicine. 2015;25(1):1-5. doi: 10.1038/npjpcrm.2015.17
43. Cramm JM, Nieboer AP. The changing nature of chronic care and coproduction of care between primary care professionals and patients with COPD and their informal caregivers. International journal of chronic obstructive pulmonary disease. 2016;11:175-82. doi: 10.2147/COPD.S94409
44. Rosso E, McGrath R. Promoting physical activity among children and youth in disadvantaged South Australian CALD communities through alternative community sport opportunities. Health Promotion Journal of Australia. 2016;27(2):105-10. doi: doi.org/10.1071/HE15092.
45. Morales-Perez A, Nava-Aguilera E, Legorreta-Soberanis J, Paredes-Solís S, Balanzar-Martínez A, Serrano-de los Santos FR, et al. Which Green Way: description of the intervention for mobilising against Aedes aegypti under difficult security conditions in southern Mexico. BMC Public Health. 2017;17(1):398. doi: 10.1186/s12889-017-4300-1
46. Revenäs Å, Forsberg HH, Granström E, Wannheden C. Co-Designing an eHealth service for the Co-Care of Parkinson disease: explorative study of values and challenges. JMIR research protocols. 2018;7(10):e11278. doi: 10.2196/11278
47. Boyd H, McKernon S, Mullin B, Old A. Improving healthcare through the use of co-design. New Zealand Medical Association. 2012;125(1357):76-87.
48. Petrakis M, Brophy L, Lewis J, Stylianou M, Scott M, Cocks N, et al. Consumer measures and research co-production: A pilot study evaluating the recovery orientation of a mental health program collaboration. Asia Pacific Journal of Social Work and Development. 2014;24(1-2):94-108. doi: 10.1080/02185385.2014.885212.
49. Ferguson M, Maidment D, Henshaw H, Gomez R. Knowledge is power: improving outcomes for patients, partners, and professionals in the digital age. Perspectives of the ASHA Special Interest Groups. 2019;4(1):140-8. doi: 10.1044/2018_PERS-SIG7-2018-0006.
50. Brosseau M, Gagnon D, Rohde K, Schellinck J. Integrating Engagement and Improvement Work in a Pediatric Hospital. Healthcare quarterly 2017;20(1):67-72. doi: 10.12927/hcq.2017.25071
51. Garzo A, Silva PA, Garay-Vitoria N, Hernandez E, Cullen S, De Cock VC, et al. Design and development of a gait training system for Parkinson's disease. PloS one. 2018;13(11). doi: 10.1371/journal.pone.0207136.
52. Davies J, Sampson M, Beesley F, Smith D, Baldwin V. An evaluation of Knowledge and Understanding Framework personality disorder awareness training: Can a co-production model be effective in a local NHS mental health Trust? Personality and mental health. 2014;8(2):161-8. doi: 10.1002/pmh.1257

For peer review only

53. Montenegro M, Greenhill B. Evaluating 'freda challenge': a coproduced human rights board game in services for people with intellectual disabilities. Journal of Applied Research in Intellectual Disabilities. 2015;28(3):223-37. doi: 10.1111/jar.12124

54. Khan A, Spector ND, Baird JD, Ashland M, Starmer AJ, Rosenbluth G, et al. Patient safety after implementation of a coproduced family centered communication programme: multicenter before and after intervention study. BMJ. 2018;363. doi: 10.1136/bmj.k4764.

55. Webb MJ, Wadley G, Sanci LA. Improving patient-centered care for young people in general practice with a codesigned screening app: mixed methods study. JMIR mHealth and uHealth. 2017;5(8):e118. doi: 10.2196/mhealth.7816

56. Haynes E, Marawili M, Marika BM, Mitchell AG, Phillips J, Bessarab D, et al. Community-based participatory action research on rheumatic heart disease in an Australian Aboriginal homeland: Evaluation of the 'On track watch'project. Evaluation and program planning. 2019;74:38-53. doi: 10.1016/j.evalprogplan.2019.02.010.

57. Rawson TM, Moore LS, Castro-Sanchez E, Charani E, Hernandez B, Alividza V, et al. Development of a patient-centred intervention to improve knowledge and understanding of antibiotic therapy in secondary care. Antimicrobial Resistance & Infection Control. 2018;7(1):1-10. doi: 10.1186/s13756-018-0333-1

58. Tsianakas V, Robert G, Richardson A, Verity R, Oakley C, Murrells T, et al. Enhancing the experience of carers in the chemotherapy outpatient setting: an exploratory randomised controlled trial to test impact, acceptability and feasibility of a complex intervention co-designed by carers and staff. Supportive Care Cancer. 2015;23(10):3069-80. doi: 10.1007/s00520-015-2677-x

59. Revenäs Å, Opava CH, Ahlén H, Brusewitz M, Pettersson S, Åsenlöf P. Mobile internet service for self-management of physical activity in people with rheumatoid arthritis: evaluation of a test version. RMD open. 2016;2(1):e000214. doi: 10.1136/rmdopen-2015-000214

60. Baxter S, Reed H, Clarke Z, Judge S, Heron N, Mccarthy A, et al. Evaluating a novel cervical orthosis, the Sheffield Support Snood, in patients with amyotrophic lateral sclerosis/motor neuron disease with neck weakness. Amyotrophic Lateral Sclerosis and Frontotemporal Degeneration. 2016;17(5-6):436-42. doi: 10.3109/21678421.2016.1148170

61. d'Young AI, Young L, Ockelford PA, Brasser M, Slavin K, Manson L, et al. The use of a co-design model in improving timely bleed reporting by adults with haemophilia living in the Auckland region of New Zealand. Clinical Haemophilia. 2014;20(3):388-97. doi: 10.1111/hae.12336

62. Eades S. Impact evaluation of an Independent Mental Health Advocacy (IMHA) service in a high secure hospital: a co-produced survey measuring self-reported changes to patient self-determination. Mental health and social inclusion. 2018. doi: 10.1108/MHSI-12-2017-0054

63. Gillard S, Foster R, Turner K. Evaluating the prosper peer-led peer support network: A participatory, coproduced evaluation. Mental Health and Social Inclusion. 2016. doi: 10.1108/MHSI-12-2015-0045

64. Henshall C, Taylor B, Goodwin L, Farre A, Jones ME, Kenyon S. Improving the quality and content of midwives' discussions with low-risk women about their options for place of birth: Co-production and evaluation of an intervention package. Midwifery. 2018;59:118-26. doi: 10.1016/j.midw.2018.01.016

65. Lucock M, Bartys S, Cupac J, Delgadillo J, Denton C, Gaines S, et al. Using Implementation Intentions to Prevent Relapse after Psychological Treatment for Depression–the SMArT Intervention. Behavioural and cognitive psychotherapy. 2018;46(5):626-32. doi: 10.1017/S1352465818000255

66. Taylor A, Dorer G, Gleeson K. Evaluation of a peer support specialist led group. Mental Health and Social Inclusion. 2018. doi: 10.1108/MHSI-03-2018-0012

67. Ogrin R, Viswanathan R, Aylen T, Wallace F, Scott J, Kumar D. Co-design of an evidence-based health education diabetes foot app to prevent serious foot complications: a feasibility study. Practical Diabetes. 2018;35(6):203-9d. doi: 10.1002/pdi.2197

68. Armitage G, Moore S, Reynolds C, Laloë P-A, Coulson C, McEachan R, et al. Patient-reported safety incidents as a new source of patient safety data: an exploratory comparative study in an acute hospital in England. Journal of health services research & policy. 2018;23(1):36-43. doi: 10.1177/1355819617727563

For peer review only

69. Sundram F, Hawken SJ, Stasiak K, Lucassen MF, Fleming T, Shepherd M, et al. Tips and traps: lessons from codesigning a clinician e-monitoring tool for computerized cognitive behavioral therapy. JMIR mental health. 2017;4(1):e3. doi: 10.2196/mental.5878
70. Farmer J, Carlisle K, Dickson-Swift V, Teasdale S, Kenny A, Taylor J, et al. Applying social innovation theory to examine how community co-designed health services develop: using a case study approach and mixed methods. BMC health services research. 2018;18(1):68. doi: 10.1186/s12913-018-2852-0
71. Litchfield IJ, Bentham LM, Lilford RJ, McManus RJ, Hill A, Greenfield S. Adaption, implementation and evaluation of collaborative service improvements in the testing and result communication process in primary care from patient and staff perspectives: a qualitative study. BMC health services research. 2017;17(1):615. doi: 10.1186/s12913-017-2566-8
72. Hackett CL, Mulvale G, Miatello A. Co-designing for quality: Creating a user-driven tool to improve quality in youth mental health services. Health Expectations. 2018;21(6):1013-23. doi: 10.1111/hex.12694
73. Levine D, Duszak R, Jr. Evolving Priorities for Imaging Utilization Research: From Outputs to Outcomes. JAMA. 2019;322(9):819-21. doi: 10.1001/jama.2019.10158.
74. Nelson EC, Batalden PB, Godfrey MM. Measurment and monitoring. In: Nelson EC, Batalden PB, Godfrey MM, editors. Quality by Design - a clinical microsystem approach: John: Bass; 2007.
75. Morse JM. Mixed method design: Principles and procedures: Routledge; 2016. ISBN: 1315424525.
76. Curry LA, Krumholz HM, O'Cathain A, Clark VLP, Cherlin E, Bradley EH. Mixed methods in biomedical and health services research. Circulation: Cardiovascular Quality and Outcomes. 2013;6(1):119-23. doi: 10.1161/CIRCOUTCOMES.112.967885
77. Marsilio M, Fusco F, Gheduzzi E, Guglielmetti C. Co-Production Performance Evaluation in Healthcare. A Systematic Review of Methods, Tools and Metrics. International journal of environmental research and public health. 2021;18(7):3336. doi: 10.3390/ijerph18073336
78. Nelson EC, Batalden PB, Godfrey MM. Quality by design: a clinical microsystems approach. San Fransisco: Jossey: Bass; 2007. ISBN: 1118046838.
79. Langley GJ, Moen R, Nolan KM, Nolan TW, Norman CL, Provost LP. The improvement guide: a practical approach to enhancing organizational performance. 2nd ed. San Fransisco: Jossey-Bass; 2009. ISBN: 9780470192412.
80. Kaplan RS, Norton DP. Linking the balanced scorecard to strategy. California management review. 1996;39(1):53-79. doi: 10.2307/41165876.
81. Greenhalgh T, Hinton L, Finlay T, Macfarlane A, Fahy N, Clyde B, et al. Frameworks for supporting patient and public involvement in research: systematic review and co-design pilot. Health expectations. 2019;22(4):785-801. doi: 10.1111/hex.12888.
82. Marks L, Laird Y, Trevena H, Smith BJ, Rowbotham S. A Scoping Review of Citizen Science Approaches in Chronic Disease Prevention. Frontiers in Public Health. 2022:953. doi: 10.3389/fpubh.2022.743348.
83. Bashir R, Surian D, Dunn AG. Time-to-update of systematic reviews relative to the availability of new evidence. Systematic Reviews. 2018;7(1):1-8. doi: 10.1186/s13643-018-0856-9.
84. Palmer VJ. The participatory zeitgeist in health care: it is time for a science of participation. Journal of Participatory Medicine. 2020;12(1):e15101.

For peer review only

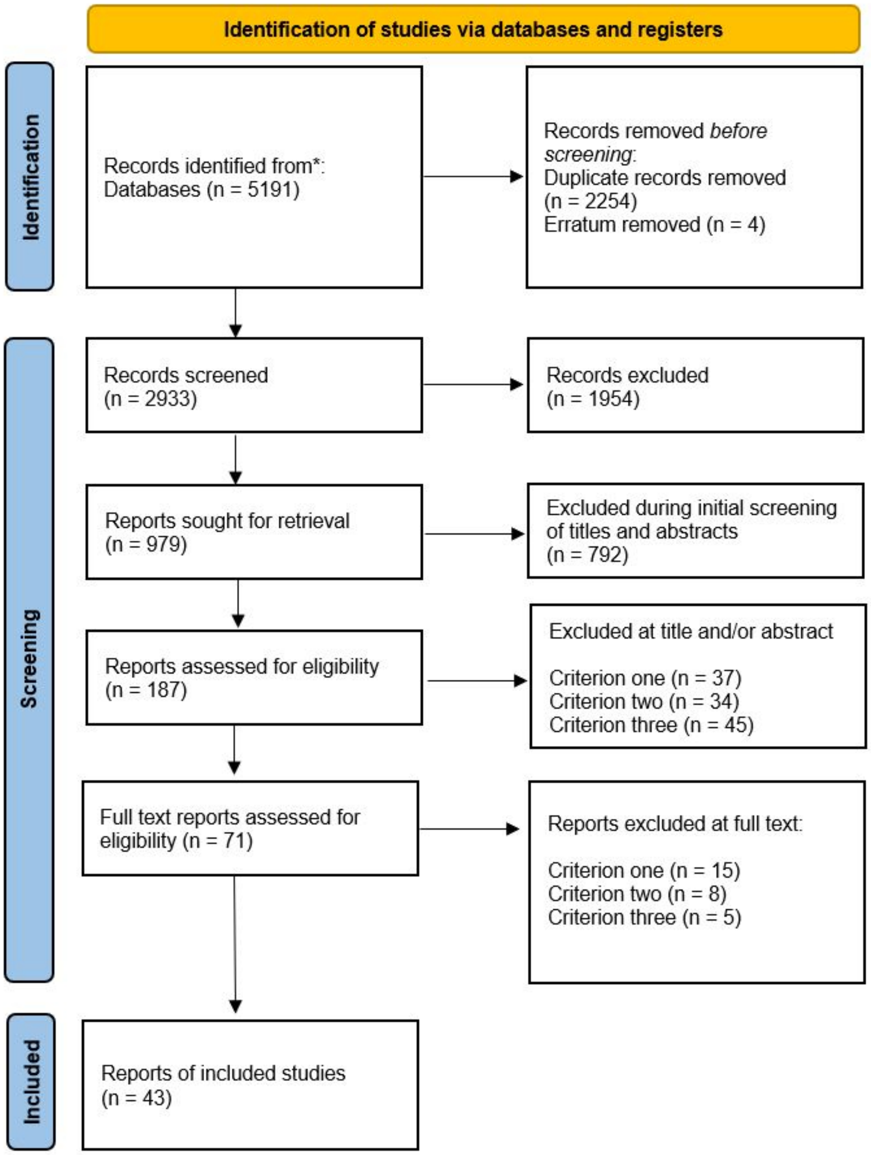

Figure 1

144x187mm (125 x 125 DPI)

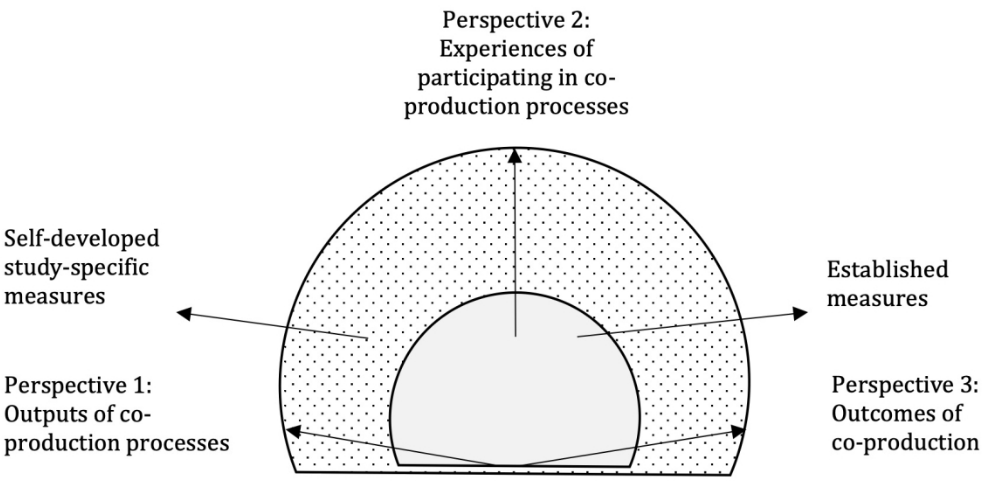

Figure 2

599x294mm (72 x 72 DPI)

The measurement and outcomes of co-production in health and social care – a systematic review of empirical studies

Supplementary file 1. Search strategy for each database

**Search terms to be included in title, abstract or key words**
(Co-produc* OR coproduc* OR co-design* OR codesign*)<br>**AND** (health OR social OR "Public Service*" OR "public sector")<br>**AND NOT** (engineer* OR biomass OR bioproduct* OR osmosis OR microalgae OR ecosystems OR hydrogen OR Brand OR Algebra)

Note: At the time of our search (March 2019) and writing, the searched databases lacked controlled vocabulary or subject headings for 'co-production', so the decision was made to only search in the title, abstract, and key word (subjects) fields. Excluding irrelevant contexts was aided through the search strategy (engineer* OR biomass OR bioproduct* OR osmosis OR microalgae OR ecosystems OR hydrogen OR Brand OR Algebra). An alternative to incorporating these terms could have been to only search subject specific databases (e.g., MEDLINE) but this may have omitted a significant number of relevant papers.

CINAHL with Full Text (EBSCOHost) – 588 hits on March 18, 2019
TI ((co-produc* OR coproduc* OR co-design* OR codesign*) AND (health OR social OR "public service*" OR "public sector*") NOT (engineer* OR biomass OR chemic* OR bioproduct* OR osmosis OR microalgae OR ecosystems OR hydrogen OR brand OR algebra)) OR AB ((co-produc* OR coproduc* OR co-design* OR codesign*) AND (health OR social OR "public service*" OR "public sector*") NOT (engineer* OR biomass OR chemic* OR bioproduct* OR osmosis OR microalgae OR ecosystems OR hydrogen OR brand OR algebra))
Limiters - Peer Reviewed; English Language

Cochrane Central Register of Controlled Trials (Wiley) – 47 hits on March 19, 2019
(co-produc* OR coproduc* OR co-design* OR codesign*):ti,ab,kw AND (health OR social OR "public service*" OR "public sector*"):ti,ab,kw NOT (engineer* OR biomass OR chemic* OR bioproduct* OR osmosis OR microalgae OR ecosystems OR hydrogen OR brand OR algebra):ti,ab,kw

MEDLINE (EBSCOHost) – 793 hits on March 18, 2019
TI ((co-produc* OR coproduc* OR co-design* OR codesign*) AND (health OR social OR "public service*" OR "public sector*") NOT (engineer* OR biomass OR chemic* OR bioproduct* OR osmosis OR microalgae OR ecosystems OR hydrogen OR brand OR algebra)) OR AB ((co-produc* OR coproduc* OR co-design* OR codesign*) AND (health OR social OR "public service*" OR "public sector*") NOT (engineer* OR biomass OR chemic* OR bioproduct* OR osmosis OR microalgae OR ecosystems OR hydrogen OR brand OR algebra))

For peer review only

Limiters – English Language

PsycINFO (ProQuest) – 403 hits on March 18, 2019
ti((co-produc* OR coproduc* OR co-design* OR codesign*) AND (health OR social OR "public service*" OR "public sector*") NOT (engineer* OR biomass OR chemic* OR bioproduct* OR osmosis OR microalgae OR ecosystems OR hydrogen OR brand OR algebra)) OR ab((co-produc* OR coproduc* OR co-design* OR codesign*) AND (health OR social OR "public service*" OR "public sector*") NOT (engineer* OR biomass OR chemic* OR bioproduct* OR osmosis OR microalgae OR ecosystems OR hydrogen OR brand OR algebra))
Limits – Peer reviewed; Language: English

PubMed (legacy) – 898 hits on March 18, 2019
Search (((co-produc*[Title/Abstract] OR coproduc*[Title/Abstract] OR co-design*[Title/Abstract] OR codesign*[Title/Abstract])) AND (health[Title/Abstract] OR social[Title/Abstract] OR “public service*”[Title/Abstract] OR “public sector*”[Title/Abstract])) NOT (engineer*[Title/Abstract] OR biomass[Title/Abstract] OR chemic*[Title/Abstract] OR bioproduct*[Title/Abstract] OR osmosis[Title/Abstract] OR microalgae[Title/Abstract] OR ecosystems[Title/Abstract] OR hydrogen[Title/Abstract] OR brand[Title/Abstract] OR algebra[Title/Abstract])
Filters: English

Scopus (Elsevier) – 2462 hits on March 18, 2019
(TITLE-ABS (co-produc* OR coproduc* OR co-design* OR codesign*) AND TITLE-ABS (health OR social OR "public service*" OR "public sector*") AND NOT TITLE-ABS (engineer* OR biomass OR chemic* OR bioproduct* OR osmosis OR microalgae OR ecosystems OR hydrogen OR brand OR algebra)) AND (LIMIT-TO (LANGUAGE , "English"))

For peer review only

Appendix 1. Studies, outcomes and measures.

Qualitative studies				
**Paper**	**International validated scales**	**Measures based on publications**	**Self-developed study-specific measures**	**Measures on the experience of co-production**
Tips and Traps: Lessons From Co-designing a Clinician E-Monitoring Tool for Computerized Cognitive Behavioral Therapy [69]				
Shaping innovations in long-term care for stroke survivors with multimorbidity through stakeholder engagement [32]				
Adaption, implementation and evaluation of collaborative service improvements in the testing and result communication process in primary care from patient and staff perspectives: a qualitative study [71]				
Applying social innovation theory to examine how community co-designed health services develop: using a case study approach and mixed methods [70]				
Co-designing for quality: Creating a user-driven tool to improve quality in youth mental health services [72]				

Note Empty fields in the table indicate that no measures were identified. In the qualitative studies, measures are not applicable.

For peer review only

Quantitative randomized control trials				
**Papers**	**International validated scales**	**Measures based on publications**	**Self-developed study-specific measures**	**Measures on the experience of co-production**
A Smartphone App (BlueIce) for Young People Who Self-Harm: Open Phase 1 Pre-Post Trial [10]	Strengths and Difficulties Questionnaire (SDQ)<br><br>Mood and Feelings Questionnaire (MFQ)<br><br>Revised Child Anxiety and Depression Scale (RCADS)		The intervention and self-harm	
Digital interventions in severe mental health problems: Lessons from the Actissist development and trial [33]	Positive and Negative Syndrome Scale (PANSS)<br><br>Calgary Depression Scale for Schizophrenia (CDSS)		The intervention and safety	
Who's challenging who training for staff empathy towards adults with challenging behaviour: cluster randomised controlled trial [35]	Maslach Burnout Inventory (MBI)<br><br>Community Living Attitudes Scale (CLAS-MR)<br><br>Staff Positive Contributions Questionnaire (SPCQ)	Staff Empathy for people with Challenging Behavior Questionnaire (SECBO)		
The impact of advertising patient and public involvement on trial recruitment: embedded cluster randomized recruitment trial [34]			Effects of the intervention	

For peer review only

Quantitative non-randomized controlled studies				
**Papers**	**International validated scales**	**Measures based on publications**	**Self-developed study-specific measures**	**Measures on the experience of co-production**
Implementing Patient-Oriented Discharge Summaries (PODS): A Multisite Pilot Across Early Adopter Hospitals [36]			Implementation process and intervention functionality	
A quality improvement initiative in community mental health in the republic of Ireland [38]			Experiences of the improvements	
Community organizing and community health: piloting an innovative approach to community engagement applied to an early intervention project in south London [37]	The General Health Questionnaire-12 (GHQ-12)<br><br>The Warwick-Edinburgh Mental Well-being Scale (WEMWBS)	Social Support Program Acceptability Rating Scale (The Treatment Acceptability Rating Scale)<br><br>Functionality of the project (NICE)<br><br>The Adapted Social Capital Questionnaire (World Bank's Social Capital Integrated Questionnaire)	Activities in the program	

For peer review only

Quantitative descriptive studies				
**Papers**	**International validated scales**	**Measures based on publications**	**Self-developed study-specific measures**	**Measures on the experience of co-production**
A co-produced self-management programme improves psychosocial outcomes for people living with depression [41]	Patient Activation Measure (PAM)<br><br>Patient Health Questionnaire (PHQ-9)<br><br>Hospital Anxiety Depression Scale (HADS)<br><br>EuroQol<br><br>Health Education Impact Questionnaire (heiQ)			
Impact of a patient-specific co-designed COPD care scorecard on COPD care quality: a quasi-experimental study [42]		Six quality indicators (NICE) matched against data from clinical records	Clinical outcomes of the change	
Health Outcomes and Patient Empowerment: The case of Health budgets in Italy [18]	The Health of the Nation Outcome Scale, Italian version (HoNOS)	Levels of co-production intensity	Costs of a program	
The changing nature of chronic care and co-production of care between primary care professionals and patients with COPD and their informal caregivers [43]	Patient Assessment of Chronic Illness Care questionnaire (PACIC)<br><br>Assessment of Chronic Illness Care, Short version (ACIC-S)			RCS to assess perceptions of interaction productivity
Can an interactive e-learning training package improve the understanding of personality disorder within mental health professionals? [40]		The Personality Disorder-Knowledge, Attitudes and Skills Questionnaire (PD-KASQ)		

For peer review only

		Efficiency of intervention (researchers' earlier study)		
Okay to Stay? A new plan to help people with long-term conditions remain in their own homes [39]			Efficiency of the program	

For peer review only

Mixed method studies				
**Papers**	**International validated scales**	**Measures based on publications**	**Self-developed study-specific measures**	**Measures on the experience of co-production**
Improving healthcare through the use of co-design [47]		Experiences of program and number of ideas. (NHS survey)		
An evaluation of Knowledge and Understanding Framework Personality Disorder Awareness Training: Can a co-production model be effective in a local NHS mental health Trust? [52]		The Personality Disorder-Knowledge, Attitudes and Skills Questionnaire (PD-KASQ)	Effects of the program	
Consumer measures and research co-production: A pilot study evaluating the recovery orientation of a mental health program collaboration [48]	The Recovery Self-Assessment (RSA)<br><br>The Recovery Enhancing Environment measure (REE)			
The use of a co-design model in improving timely bleed reporting by adults with haemophilia living in the Auckland region of New Zealand [61]		Mean bleed reporting time<br><br>Bleeds per month<br><br>Bleeding episodes outside of a prespecified 48-h	The clinical effects of the program	
Evaluating 'FREDA Challenge': A co-produced human rights board game in services for people with intellectual disabilities [53]		Human right quiz<br><br>Attitudes to Human Rights questionnaire (ATHIRI)	The attitudes, knowledge, and opinions of the intervention	
Evaluating a novel cervical orthosis, the Sheffield Support Snood, in patients with amyotrophic lateral sclerosis/motor neuron disease with neck weakness [60]			The experiences, comfort and satisfaction with the intervention	
Evaluating the Prosper peer-led peer support network: A participatory, coproduced evaluation [63]			The experiences and priorities on the service	

For peer review only

Mobile internet service for self-management of physical activity in people with rheumatoid arthritis: evaluation of a test version [59]			The innovation, self-monitoring and feedback	
Promoting physical activity among children and youth in disadvantaged South Australian CALD communities through alternative community sport opportunities [44]			The thoughts and feelings of the project	
Enhancing the experience of carers in the chemotherapy outpatient setting: an exploratory randomised controlled trial to test impact, acceptability and feasibility of a complex intervention co-designed by carers and staff [58]	The General Health Questionnaire-12 (GHQ-12)<br><br>Supportive Care Needs Survey for Partners and Carers [SCNS-P&C)	Perceived confidence in supporting friend or relative	The effects of the intervention; knowledge and experiences of care	
Improving Patient-Centered Care for Young People in General Practice With a Co-designed Screening App: Mixed Methods Study [55]	Mobile Application Rating Scale (MARS)	Patient-centered care<br><br>Disclosure<br>Fear of judgment<br>App acceptability<br><br>Knowledge, confidence and enthusiasm<br><br>Youth friendly care	The opinions and willingness to use the innovation	
Integrating Engagement and Improvement Work In a Paediatric Hospital [50]			Impressions and feelings about integrating EBCD and quality improvement tools	
Which green way: description of the intervention for mobilising against Aedes aegypti under difficult security conditions in southern Mexico [45]		Clinical indicators<br><br>Proved infections	Self-reported health outcomes	
Impact evaluation of an Independent Mental Health Advocacy (IMHA) service in a high secure hospital: a co-produced survey measuring self-reported changes to patient self-determination [62]			The improved services' relevance, accessibility and ease of use	

For peer review only

Improving the quality and content of midwives' discussions with low-risk women about their options for place of birth: Co-production and evaluation of an intervention package [64]			The level of knowledge and confidence regarding safety and intervention rates, views of the intervention package<br><br>Objective knowledge calculated using multiple-choice questions	
Design and development of a gait training system for Parkinson's disease [51]	System Usability Scale (SUS)		The development and improvement of a customer focused innovation	
Patient safety after implementation of a co-produced family centered communication programme: multicenter before and after intervention study [54]		Medical errors<br><br>Harmful errors<br><br>Adverse events	Family experiences	
Co-design of an evidence-based health education diabetes foot app to prevent serious foot complications: a feasibility study [67]	The Nottingham Assessment of Functional Footcare (NAFF)	Diabetes foot care knowledge<br><br>Attitudes		
Evaluation of a peer support specialist led group [66]			The experiences and effects of the recovery group<br><br>The number of referrals and contacts	
Patient-reported safety incidents as a new source of patient safety data: an exploratory comparative study in an acute hospital in England [68]			The number of safety incidents counted and compared by methods	
Using Implementation Intentions to Prevent Relapse after Psychological Treatment for Depression - the SMArT Intervention [65]	Patient Health Questionnaire (PHQ-9)<br><br>Generalized Anxiety Disorder Questionnaire (GAD-7)		Counts of self-regulatory strategies, IMPS	

For peer review only

Development of a patient-centred intervention to improve knowledge and understanding of antibiotic therapy in secondary care [57]		Patient knowledge and understanding surrounding antimicrobial therapy and resistance		
Co-Designing an eHealth Service for the Co-Care of Parkinson Disease: Explorative Study of Values and Challenges [46]			The experiences and views of the workshops and logistics	Experience: Questionnaire on participants' overall experiences of participation
Knowledge Is Power: Improving Outcomes for Patients, Partners, and Professionals in the Digital Age [49]	Glasgow Hearing Aid Benefit Profile (GHABP)<br><br>The International Outcome Inventory for Hearing Aids (IOI-HA)<br><br>The Hearing Handicap Inventory for the Elderly (HHIE)		Patient feedback on how they experienced the innovation	
Community-based participatory action research on rheumatic heart disease in an Australian Aboriginal homeland: Evaluation of the 'On track watch' project [56]		Values and Ethics Guidelines, National Health & Medical Research Council<br><br>'How-to' guide, National Health Medical Research Council		Emoticon survey based on National Health and Medical Research Council principles<br>Reciprocity, respect<br>Equality<br>Responsibility<br>Survival and Protection<br>Spirit & integrity

For peer review only

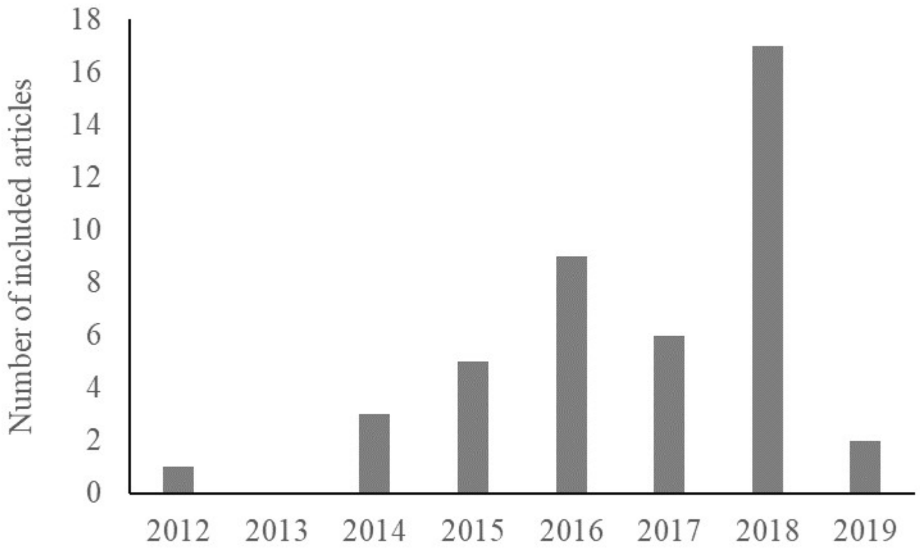

For peer review only

PRISMA 2020 Checklist for: The measurement and outcomes of co-production on health and social care – a systematic review of empirical studies

Section and Topic	Item #	Checklist item	Location where item is reported
**TITLE**			
Title	1	Identify the report as a systematic review.	Title
**ABSTRACT**			
Abstract	2	See the PRISMA 2020 for Abstracts checklist.	Abstract
**INTRODUCTION**			
Rationale	3	Describe the rationale for the review in the context of existing knowledge.	Introduction Sections starting with "There are several plausible explanations related" and "There are a small number of reviews…"
Objectives	4	Provide an explicit statement of the objective(s) or question(s) the review addresses.	Last section of the introduction.
**METHODS**			
Eligibility criteria	5	Specify the inclusion and exclusion criteria for the review and how studies were grouped for the syntheses.	The subsections Information sources, eligibility criteria, search strategy and selection process for the scoping review and Eligibility criteria and selection process for this systematic review.
Information sources	6	Specify all databases, registers, websites, organisations, reference lists and other sources searched or consulted to identify studies. Specify the date when each source was last searched or consulted.	Supplemental File 1.
Search strategy	7	Present the full search strategies for all databases, registers and websites, including any filters and limits used.	Supplemental File 1.
Selection process	8	Specify the methods used to decide whether a study met the inclusion criteria of the review, including how many reviewers screened each record and each report retrieved, whether they worked independently, and if applicable, details of automation tools used in the process.	The subsections Information sources, eligibility criteria, search strategy and selection process for the scoping review and Eligibility criteria and selection process for this systematic review.
Data collection process	9	Specify the methods used to collect data from reports, including how many reviewers collected data from each report, whether they worked independently, any processes for obtaining or confirming data from study investigators, and if applicable, details of automation tools used in the process.	Subsections Eligibility criteria and selection process for this systematic review.
Data items	10a	List and define all outcomes for which data were sought. Specify whether all results that were compatible with each outcome domain in each study were sought (e.g., for all measures, time points, analyses), and if not, the methods used to decide which results to collect.	Table 1 and Synthesis methods.
	10b	List and define all other variables for which data were sought (e.g., participant and intervention characteristics, funding sources). Describe any assumptions made about any missing or unclear information.	Table 2 and Synthesis methods.
Study risk of bias assessment	11	Specify the methods used to assess risk of bias in the included studies, including details of the tool(s) used, how many reviewers assessed each study and whether they worked independently, and if applicable, details of automation tools used in the process.	Subsections Eligibility criteria and selection process for this systematic review.
Effect measures	12	Specify for each outcome the effect measure(s) (e.g., risk ratio, mean difference) used in the synthesis or presentation of results.	N/A

For peer review only

PRISMA 2020 Checklist for: The measurement and outcomes of co-production on health and social care – a systematic review of empirical studies

Section and Topic	Item #	Checklist item	Location where item is reported
Synthesis methods	13a	Describe the processes used to decide which studies were eligible for each synthesis (e.g., tabulating the study intervention characteristics and comparing against the planned groups for each synthesis (item #5)).	Subsections Data collection process ("To develop a shared work procedure…") and Data items for this systematic review.
	13b	Describe any methods required to prepare the data for presentation or synthesis, such as handling of missing summary statistics, or data conversions.	Subsections Data collection process and data items for this systematic review and Synthesis methods.
	13c	Describe any methods used to tabulate or visually display results of individual studies and syntheses.	Table 3.
	13d	Describe any methods used to synthesize results and provide a rationale for the choice(s). If meta-analysis was performed, describe the model(s), method(s) to identify the presence and extent of statistical heterogeneity, and software package(s) used.	Subsection Synthesis methods.
	13e	Describe any methods used to explore possible causes of heterogeneity among study results (e.g., subgroup analysis, meta-regression).	N/A
	13f	Describe any sensitivity analyses conducted to assess robustness of the synthesized results.	N/A
Reporting bias assessment	14	Describe any methods used to assess risk of bias due to missing results in a synthesis (arising from reporting biases).	Subsection Strengths and limitations.
Certainty assessment	15	Describe any methods used to assess certainty (or confidence) in the body of evidence for an outcome.	N/A
**RESULTS**			
Study selection	16a	Describe the results of the search and selection process, from the number of records identified in the search to the number of studies included in the review, ideally using a flow diagram.	Subsection Study selection and Figure 1.
	16b	Cite studies that might appear to meet the inclusion criteria, but which were excluded, and explain why they were excluded.	N/A
Study characteristics	17	Cite each included study and present its characteristics.	Subsection Study characteristics and Appendix 1.
Risk of bias in studies	18	Present assessments of risk of bias for each included study.	N/A
Results of individual studies	19	For all outcomes, present, for each study: (a) summary statistics for each group (where appropriate) and (b) an effect estimate and its precision (e.g. confidence/credible interval), ideally using structured tables or plots.	N/A
Results of syntheses	20a	For each synthesis, briefly summarise the characteristics and risk of bias among contributing studies.	N/A
	20b	Present results of all statistical syntheses conducted. If meta-analysis was done, present for each the summary estimate and its precision (e.g., confidence/credible interval) and measures of statistical heterogeneity. If comparing groups, describe the direction of the effect.	N/A
	20c	Present results of all investigations of possible causes of heterogeneity among study results.	N/A
	20d	Present results of all sensitivity analyses conducted to assess the robustness of the synthesized results.	N/A
Reporting biases	21	Present assessments of risk of bias due to missing results (arising from reporting biases) for each synthesis assessed.	N/A
Certainty of evidence	22	Present assessments of certainty (or confidence) in the body of evidence for each outcome assessed.	N/A
**DISCUSSION**			
Discussion	23a	Provide a general interpretation of the results in the context of other evidence.	Subsection General interpretation of results.
	23b	Discuss any limitations of the evidence included in the review.	Subsection Strengths and limitations.
	23c	Discuss any limitations of the review processes used.	Subsection Strengths and limitations.

For peer review only - http://bmjopen.bmj.com/site/about/guidelines.xhtml

PRISMA 2020 Checklist for: The measurement and outcomes of co-production on health and social care – a systematic review of empirical studies

Section and Topic	Item #	Checklist item	Location where item is reported
	23d	Discuss implications of the results for practice, policy, and future research.	Subsections Implications for practice and Implications for future research.
**OTHER INFORMATION**			
Registration and protocol	24a	Provide registration information for the review, including register name and registration number, or state that the review was not registered.	Introduction. Section starting "In the published Samskapa study protocol…"
	24b	Indicate where the review protocol can be accessed, or state that a protocol was not prepared.	Introduction.
	24c	Describe and explain any amendments to information provided at registration or in the protocol.	Subsection Strengths and limitations.
Support	25	Describe sources of financial or non-financial support for the review, and the role of the funders or sponsors in the review.	Subsection Funding.
Competing interests	26	Declare any competing interests of review authors.	Subsection Conflict of interest.
Availability of data, code and other materials	27	Report which of the following are publicly available and where they can be found: template data collection forms; data extracted from included studies; data used for all analyses; analytic code; any other materials used in the review.	None*

'* Research Data at the School of Health and Welfare is regulated under the Freedom of the Press Act (1949:105) and the Public Access to Information and Security Act (2009:4) as Public Records/Official Documents. Research data that is registered and Archived at the School of Health and Welfare can be requested anonymously by anyone for a fixed fee according to the Fee Regulation (1992:191). If the research data contain sensitive information, like personal data and/or trade secrets, that information is protected by Confidentiality and Secrecy by the Public Access to Information and Security Act and requests of such information will be denied with the possibility to appeal the denial in the Court of Appeals. Secret or Confidential research data can be accessed by other researchers if they receive permission from the Regional Ethics Review Board. The lawful basis for transmission of Secret and/or Confidential information is then based on the Law of Ethics Review for Research on Humans (2003:460)

For peer review only
